# Genetic signature to provide robust risk assessment of psoriatic arthritis development in psoriasis patients

**DOI:** 10.1038/s41467-018-06672-6

**Published:** 2018-10-09

**Authors:** Matthew T. Patrick, Philip E. Stuart, Kalpana Raja, Johann E. Gudjonsson, Trilokraj Tejasvi, Jingjing Yang, Vinod Chandran, Sayantan Das, Kristina Callis-Duffin, Eva Ellinghaus, Charlotta Enerbäck, Tõnu Esko, Andre Franke, Hyun M. Kang, Gerald G. Krueger, Henry W. Lim, Proton Rahman, Cheryl F. Rosen, Stephan Weidinger, Michael Weichenthal, Xiaoquan Wen, John J. Voorhees, Gonçalo R. Abecasis, Dafna D. Gladman, Rajan P. Nair, James T. Elder, Lam C. Tsoi

**Affiliations:** 1 Department of Dermatology, University of Michigan Medical School, Ann Arbor, 48109 MI USA; 2 Morgridge Institute for Research, Madison, 53715 WI USA; 3 Ann Arbor Veterans Affairs Hospital, Ann Arbor, 48105 MI USA; 4 Department of Biostatistics, Center for Statistical Genetics, University of Michigan, Ann Arbor, 48109 MI USA; 5 Department of Human Genetics, Emory University School of Medicine, Atlanta, 30322 GA USA; 6 Department of Medicine, Division of Rheumatology, University of Toronto, Toronto Ontario, M5G 2C4 Canada; 7 Centre for Prognosis Studies in the Rheumatic Diseases, Krembil Research Institute, University of Toronto, Toronto Ontario, M5T 2S8 Canada; 8 Institute of Medical Science, University of Toronto, Toronto Ontario, M5S 1A8 Canada; 9 Department of Laboratory Medicine and Pathobiology, University of Toronto, Toronto Ontario, M5S 1A8 Canada; 10 Department of Dermatology, University of Utah, Salt Lake City, Utah 84132 USA; 11 Institute of Clinical Molecular Biology, Christian-Albrechts-University of Kiel, Kiel, 24105 Germany; 12 Department of Dermatology, Linköping University, Linköping, SE-581 83 Sweden; 13 Estonian Genome Center, University of Tartu, Tartu, 51010 Estonia; 14 Broad Institute of MIT and Harvard, Cambridge, Massachusetts 02142 USA; 15 Department of Dermatology, Henry Ford Hospital, Detroit, 48202 MI USA; 16 Memorial University, St. John’s, Newfoundland and Labrador A1B 3X9 Canada; 17 Division of Dermatology, Toronto Western Hospital, University of Toronto, Toronto, M5G 2C4 Ontario Canada; 18 Department of Dermatology, University Medical Center Schleswig-Holstein, Campus Kiel Kiel, 24105 Germany; 19 Department of Computational Medicine & Bioinformatics, University of Michigan, Ann Arbor, 4810 MI USA

## Abstract

Psoriatic arthritis (PsA) is a complex chronic musculoskeletal condition that occurs in ~30% of psoriasis patients. Currently, no systematic strategy is available that utilizes the differences in genetic architecture between PsA and cutaneous-only psoriasis (PsC) to assess PsA risk before symptoms appear. Here, we introduce a computational pipeline for predicting PsA among psoriasis patients using data from six cohorts with >7000 genotyped PsA and PsC patients. We identify 9 new loci for psoriasis or its subtypes and achieve 0.82 area under the receiver operator curve in distinguishing PsA vs. PsC when using 200 genetic markers. Among the top 5% of our PsA prediction we achieve >90% precision with 100% specificity and 16% recall for predicting PsA among psoriatic patients, using conditional inference forest or shrinkage discriminant analysis. Combining statistical and machine-learning techniques, we show that the underlying genetic differences between psoriasis subtypes can be used for individualized subtype risk assessment.

## Abstract (web summary)

Approximately 30% of psoriasis patients develop psoriatic arthritis (PsA) and early diagnosis is crucial for the management of PsA. Here, Patrick et al. develop a computational pipeline involving statistical and machine-learning methods that can assess the risk of progression to PsA based on genetic markers.

## Introduction

Psoriatic arthritis (PsA) is a chronic inflammatory musculoskeletal condition associated with psoriasis vulgaris (PsV) that affects populations of people worldwide. Although the prevalence of PsA is rare in the general population (<0.5%)^1^, it occurs in ~30% of psoriasis patients^2^, and its symptoms (joint pain, swelling, and limitation of movement and deformity) typically arise after psoriasis has been diagnosed based on skin lesions^3^. PsA has been shown to cause reduced quality of life and is associated with comorbidities that increase mortality^4^, thus posing a significant social and economic burden to society. Early diagnosis is critical for effective management, and the longer symptoms continue before being diagnosed, the worse the outcome typically is^5^; a delay of 6 months, until consultation with a rheumatologist, was found to result in more severe joint erosion and inflammation^6^. It is difficult to diagnose PsA early because of variation in the way the disease manifests itself and how it develops^7^; in a recent meta-analysis^8^, 15% of psoriasis patients undergoing dermatology treatment/monitoring were estimated to have undiagnosed PsA. Current approaches to PsA diagnosis are based on clinical, laboratory and radiological features^9^, including the use of criteria such as ClASsification criteria for Psoriatic ARthritis^10^ and MAdrid Sonographic Enthesitis Index^11^. However, there is limited systematic strategy to provide quantitative assessment for PsA risk among psoriasis patients, before symptoms appear.

The heritability of PsA is estimated to be around 80%^12^, higher than that reported for psoriasis in general. While this suggests a genetic risk metric should be achievable, PsA shares many of the same genetic loci as cutaneous-only psoriasis (PsC)^13^, patients who have PsV but do not get PsA. The development of a PsA-risk assessment metric is therefore far from trivial. Genetic difference has been observed between PsA and PsC in the major histocompatibility complex (MHC)^14,15^, and other loci have also presented genetic heterogeneity. However, only variants in the MHC region have so far been found to distinguish PsA from PsC with genome-wide significance^13^. In fact, due to the subtle genetic differences between PsA and PsC, large sample sizes are required to provide sufficient statistical power to identify signals that differentiate the two subtypes. While early GWAS were limited by the number of genotyped patients available with subtype information^16–18^, recent international collaborations^13,19–22^ have enabled us to collect more PsA and PsC samples.

In this study, we hypothesize that by combining statistical genetics and machine-learning approaches, we would be able to assess the risk of PsA (and PsC) among psoriasis patients using genetic information. Compared to a previous genetic study on these two psoriasis subtypes^13^, our study significantly increases the number of samples with genome-wide content. As a result, our study identifies one new genome-wide significant locus for psoriasis and further reveal eight new loci for psoriasis subtypes. We also show that the genetic differences between the two subtypes are enriched in regulatory elements of lymphocytes. Machine-learning techniques (including random forest, conditional inference forest, shrinkage discriminant analysis, and elastic net regression) are applied to predict PsA and PsC status from these differences. While success has previously been achieved using machine-learning to distinguish subtypes of inflammatory bowel disease^23^, we here attempt to apply these techniques to evaluate the genetic risk of psoriasis subtypes. We show that genetic information can be used to classify subtypes among psoriasis patients (AUC = 0.82), with the ability to accurately predict psoriasis subtypes especially among individuals carrying the most extreme genetic burden (e.g., we achieve over 90% precision for the top 5% of patients predicted as having PsA).

## Results

### Overview

**Fig. 1:**
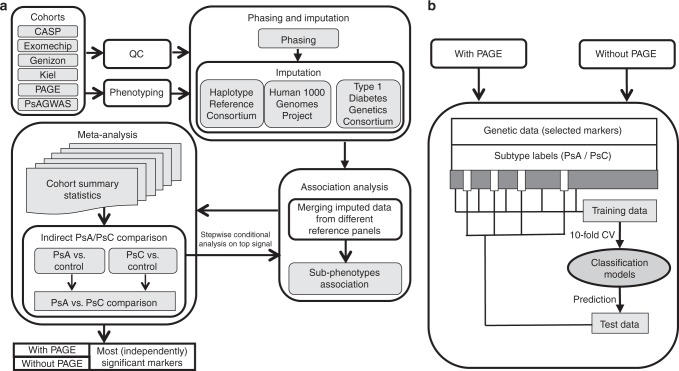
Computational pipeline to predict psoriasis subtypes. **a** Overview of pipeline, through quality control, phasing and imputation, association analysis, meta-analysis, and stepwise conditional analysis. **b** The machine-learning process included separating data randomly into training (cross-validation to optimize the model) and test (holdout) sets, as well as evaluating the results with and without the PAGE Immunochip dataset. PsA psoriatic arthritis; PsC cutaneous-only psoriasis; QC quality control

Our pipeline for predicting psoriasis subtypes involves five stages (Fig. 1a): data processing (quality control and rephenotyping), phasing and genotype/amino acid imputation, association analyses per cohort, meta-analyses, and machine-learning for subtype prediction and risk assessment. After data processing and imputation, we conducted cohort-specific association analysis and meta-analysis iteratively, to select independent markers differentiating the subtypes (PsA and PsC); finally, we utilized machine-learning techniques to build models for subtype risk assessment (Fig. 1b).

### Numbers of samples and markers enhanced

To provide a comprehensive evaluation of the genetic differences between PsA and PsC, we included genotyped samples from five GWAS datasets (CASP^19^, Exomechip with GWAS content^20^, Genizon^21^, Kiel^21^, and PsA GWAS^13^) and one Immunochip dataset (PAGE^22^). The total numbers of PsA and PsC samples were increased by 20 and 15% (Table 1), among which PsA and PsC samples with GWAS coverage were increased, respectively by 39% and 97% (to 2703 PsA and 2681 PsC samples), compared to the previous largest meta-analysis of psoriasis subtypes^13^. This was achieved by including an additional cohort, Exomechip (which contains genome-wide markers for over 10,000 samples) and rephenotyping the samples to incorporate recent PsA diagnoses. The density and diversity of genetic/amino acid markers were raised substantially through genetic and HLA imputation and by combining the Haplotype Reference Consortium (HRC)^24^ and 1000 Genomes Project (1KG)^25^ as reference panels: we increased the number of well-imputed (i.e., *r*^2^ ≥ 0.7) single nucleotide polymorphisms (SNPs) and short insertions and deletions (INDELs) for each cohort by 12–17% (Supplementary Table 1), when compared to using either one of the panels alone. To evaluate the quality of our imputation, we compared the imputation results with genotyped data for 24 independent markers previously genotyped in 6052 samples from CASP, PsAGWAS, and PAGE^13^. In most cases the observed imputation quality was higher than that predicted by Minimac, suggesting the quality of our imputation is high (Supplementary Figure 1). We then compared the imputation quality for these genotyped markers with the 200 markers we used for classification and did not observe any strong evidence of differences in imputation quality (two sided Wilcoxon test: *p* > 0.05) when including or excluding PAGE (Supplementary Figures 2 and 3). Together with previous imputation work illustrating the robustness of MaCH/Minimac genetic imputation^26^, especially for markers with high imputation quality (we used *r*^2^ ≥ 0.7 in our study), we are confident the imputed dosages reflect the actual genotypes accurately. We also performed imputation on HLA alleles/amino acid markers, using a modified version of SN2HLA. Altogether, we utilized ~9.7 million well-imputed markers with ≥1% minor allele frequencies in our association study (Table 1).

**Table 1:** Number of patients and markers in each Genetic Cohort

Cohort	Patients				Markers (genotyped and well-imputed)				
	PsV	PsA	PsC	Control	Genotyped	SNP^a^	INDEL^a^	HLA/AA^a^	Total
PsA GWAS	1430	1430	NA	1417	972,453	17,510,941	1,278,891	1251	18,791,083
CASP GWAS	1338	349	639	1370	438,609	15,759,031	1,063,919	1247	16,824,197
Kiel GWAS	464	33	269	1135	504,625	13,315,820	1,077,158	1236	14,394,214
Genizon GWAS	760	139	399	993	489,501	13,624,904	1,093,913	1224	14,720,041
Exomechip	3863	752	1374	4027	461,092	16,411,455	976,233	1254	17,388,942
PAGE Immunochip	3169	971	885	7394	160,228	1,414,274	84,270	1245	1,499,789
New Total	11,024	3674	3566	16,336	New Union	23,657,701 (8,730,264^b^)	1,403,045 (1,021,305^b^)	1270 (1217^b^)	25,062,016 (9,752,786^b^)
New GWAS Total	7855	2703	2681	8943	New intersection (All)	1,120,138 (43,356^c^)	66,845 (3301^c^)	1203 (546^c^)	1,188,186 (47,203^c^)
Previous^13^ Total	9293	3061	3110	17,393	New intersection (GWAS)	9,771,987 (247,740^c^)	870,338 (27,115^c^)	1205 (546^c^)	10,643,530 (275,401^c^)
Previous^13^ GWAS Total	4007	1946	1363	4934	Previous^13,14^ Union	8,265,477 (7,091,979^b^)	681,304 (627,111^b^)	1342 (1216^b^)	8,948,123 (7,720,306^b^)
					Previous^13,14^ intersection (All)	40,249 (8,775^c^)	3187 (717^c^)	1141 (309^c^)	44,577 (9801^c^)
					Previous^13,14^ intersection (GWAS)	6,964,145 (229,722^c^)	589,032 (20,195^c^)	1269 (326^c^)	7,554,446 (250,243^c^)

*PsV* psoriasis vulgaris; *PsA* psoriatic arthritis; *PsC* cutaneous-only psoriasis; *NA* not available

^a^Well-imputed markers (*r*^2^ ≥ 0.7)

^b^Union of markers filtered using MAF ≥ 0.01 (these are the markers used in our unconditional meta-analysis)

^c^Intersection of markers filtered using MAF ≥ 0.01 and *p* ≤ 0.05 (these are the markers used in our conditional meta-analysis). All the samples are of Caucasian descent

### New loci identified for psoriasis and psoriasis subtypes

Association analysis was performed with four different comparisons for each cohort: PsV vs. Control; PsA vs. Control; PsC vs. Control; and PsA vs. PsC. In our PsV vs. control meta-analysis, we identified a new psoriasis susceptibility locus at 13q14.2 (rs9591325; *p* = 7 × 10^−9^ [Wald test]; odds ratio = 1.25), which is located inside an intron of *DLEU1* (Supplementary Figure 4). Interestingly, the effect size of this locus is larger than those of the psoriasis loci recently identified through Immunochip GWAS-based meta-analysis^20,22,27^. Upon investigation, we found this marker was not well-imputed in the large Immunochip dataset^27^ when using 1KG as a reference panel, and thus the previous association results for this marker relied solely on the GWAS datasets. The HRC imputation panel we employed here has significantly enhanced the imputation quality of this marker (from *r*^2^ = 0.54 to 0.84), thus allowing the inclusion of the Immunochip cohort and the increase of statistical power at this locus. Interestingly, this marker was previously identified as genome-wide significant (*p* = 1 × 10^−10^)^28^ for primary biliary cirrhosis; and it was suggested to be a secondary signal for multiple sclerosis (*p* = 2 × 10^−7^)^29^, independent of the genome-wide significant primary signals (rs806349^30^: ld-*r*^*2*^ = 0.076, rs2812197^29^: ld-*r*^*2*^ = 0.14).

**Fig. 2:**
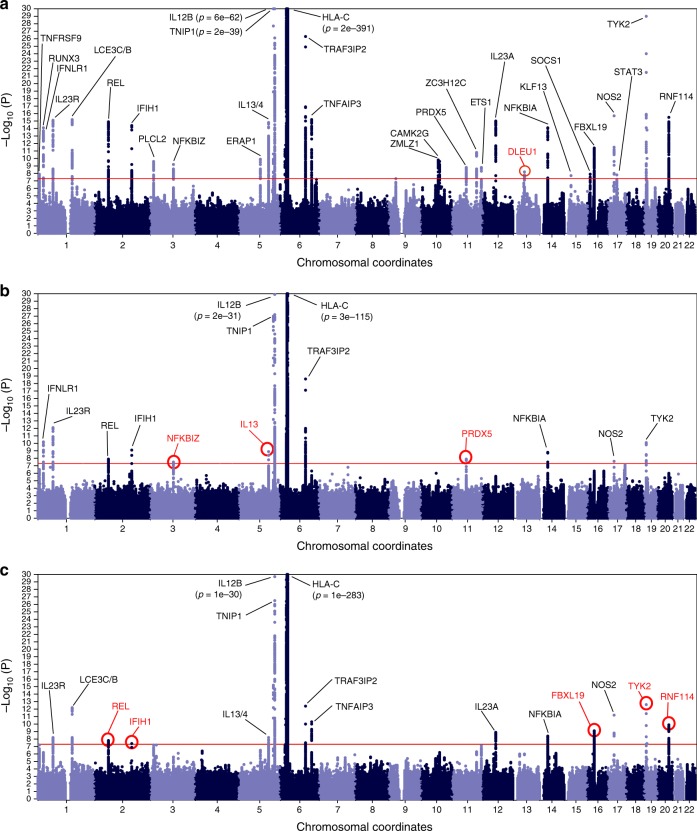
Meta-analysis results. New loci identified by this study are highlighted in red, whereas loci identified in a previous study that were not genome-wide significant in this study are highlighted in blue, for meta-analysis results based on the following comparisons: **a** PsV vs. Control; **b** PsA vs. Control; and **c** PsC vs. Control

With enhanced subphenotype sample size and number of well-imputed markers, our PsA/PsC vs. control meta-analyses showed that all 10 PsA, and 10 out of 12 PsC loci identified in the previous study^13^ still achieve genome-wide significance (*p* ≤ 5 × 10^−8^) (Fig. 2). In addition, we showed eight new genome-wide significant loci for PsA/PsC (three for PsA; five for PsC; Table 2) from PsV loci with previously unknown subtype association. Since one of the cohorts (PsA GWAS) only contains PsA samples, it was not possible to include this cohort in a direct meta-analysis comparing PsA vs. PsC. Confirming previous findings^13^, we found indirect meta-analysis (i.e., comparing summary statistics from PsA vs. control to PsC vs. control) using all the cohorts to be more powerful at differentiating the genetic architectures than direct meta-analysis without the PsA GWAS (Supplementary Figure 5). However, comparisons of the genetic architecture between PsA and PsC (direct and indirect PsA vs. PsC meta-analyses) only identified markers within MHC (Supplementary Figure 6) as having genome-wide significance, which is in concordance with our previous study^13^.

**Table 2:** Meta-analysis results for possible new psoriasis loci

Marker ID	Chr	Position (hg19)^a^	Alleles (risk/nonrisk)	Nearby gene	Phenotype comparison	Direction (PCKGEI)^b^	Control AF^c^	Case AF^c^	Meta OR^d^	Meta *p*^d^
rs9591325	13	50811220	T/C	*DLEU1*	PsV-ctl	++++++	0.921	0.934	1.25	7 × 10^−9^
rs7612823	3	101613923	T/C	*NFKBIZ*	PsA-ctl	++++++	0.806	0.836	1.25	3 × 10^−8^
rs848	5	131996500	C/A	*IL13*	PsA-ctl	++++++	0.787	0.827	1.27	1 × 10^−9^
rs588177	11	64024056	C/A	*PRDX5*	PsA-ctl	++−+++	0.301	0.339	1.20	1 × 10^−8^
rs1177202	2	61074576	C/G	*REL*	PsC-ctl	++++++	0.566	0.606	1.18	2 × 10^−8^
rs2111485	2	163110536	G/A	*IFIH1*	PsC-ctl	+++++	0.605	0.641	1.18	4 × 10^−8^
rs14990525	16	31006289	TGGTGCTA/-	*FBXL19*	PsC-ctl	+++++	0.362	0.402	1.20	9 × 10^−10^
rs34536443	19	10463118	G/C	*TYK2*	PsC-ctl	?++++	0.955	0.978	2.08	2 × 10^−13^
rs34685920	20	48572650	A/−	*RNF114*	PsC-ctl	+++++	0.568	0.608	1.20	1 × 10^−10^

Chr chromosome, AF allele frequency, OR odds ratio, *p* *p* value

^a^For insertions or deletions of the reference sequence, position of first base before the insertion point or of first base of the deleted sequence is shown, respectively

^b^For six studies of discovery meta-analysis (P = PsA GWAS, C = CASP GWAS, K = Kiel GWAS, G = Genizon GWAS, E = Exomechip, I = PAGE Immunochip) indicates whether OR of risk allele is ≥1 (+), <1 (−), or undetermined due to low imputation quality (?). PsA GWAS directions are only included for PsV-ctl and PsA-ctl, since the PsA GWAS cohort does not contain any patients with PsC subphenotype

^c^AFs are represented according to the risk allele

^d^OR and *p* value for fixed effects meta-analysis with inverse variance weighting

**Fig. 3:**
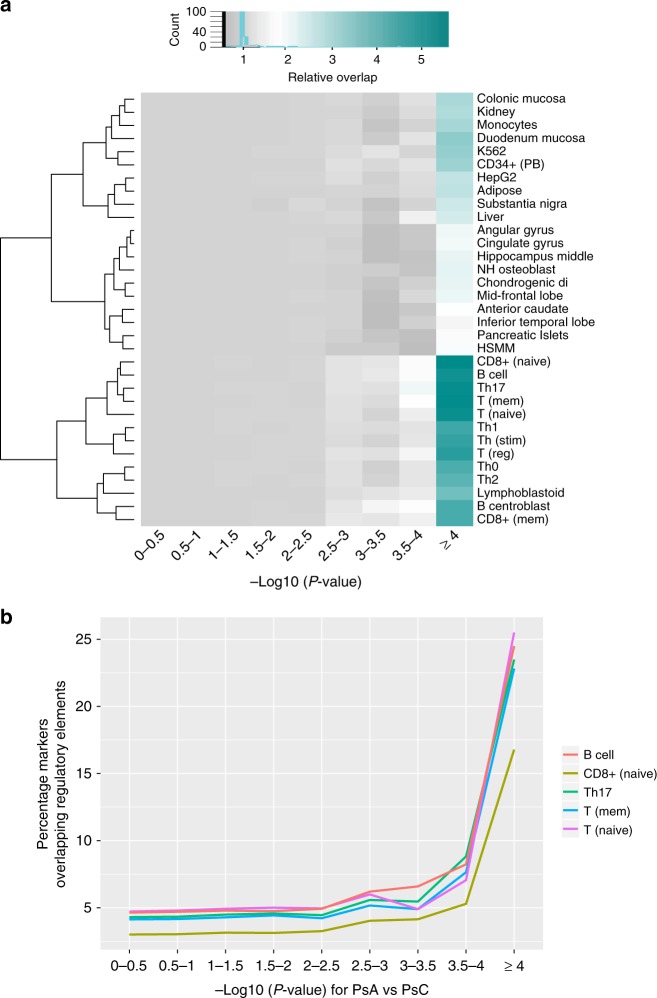
Enrichment for regulatory elements. Enrichment calculated using active enhancers predicted using H3K27ac: **a** heatmap showing the relative overlap (compared to the 0–0.05 bin) with each cell type for markers with different *p* values (from gray: no overlap, through to dark green: high overlap) and **b** overlap for the top five cell types (with highest relative increase from the baseline percentage)

Previous studies have illustrated that psoriasis loci are enriched among regulatory elements^20,31^. Here, our enrichment analysis showed markers differentiating PsA and PsC were also enriched among regulatory elements. We evaluated the proportions of overlap with active regulatory elements (measured by H3K27ac marks^31^) for markers with different significance levels in PsA vs. PsC indirect meta-analysis (see Methods). Interestingly, we found that markers with the most significant *p* values differentiating the two subtypes were enriched for H3K27ac peaks across 34 cell types we examined (Fig. 3a). Specifically, the most significant genetic markers exhibited higher overlap with active elements in immune cells. Five cell types, i.e., B-cells (adult CD20), T_naive_ (CD25- CD45RA + naive), T_memory_ (CD25− CD45RO + mem), Th_17_ (CD25− IL17 + Th17 stim), and CD8+_naive_ (CD45RA + CD8), achieve over 15% of overlap among the most significant markers (Fig. 3b). It is worth noting the MHC plays a large role in psoriasis immunology, and that outside this region, the cell type overlap is different.

### Conditional analysis for feature selection

We performed stepwise conditional analysis to identify independent features associated with psoriasis subtypes (and used for the subtype classification): we conditioned on the most significant marker (out of markers with *p* value ≤ 0.05 in the unconditional indirect PsA vs. PsC meta-analysis), and repeated the association analysis iteratively, assessing the ability of the identified features to classify the psoriasis subtypes (Fig. 1a). The results of the conditional meta-analysis were integrated with our machine-learning approach. We stopped this process once the median area under the receiver operator curve (on our cross-validation set), measured over nonoverlapping sets of ten consecutive markers (identified through conditional analysis), increased by less than 0.2% three times in a row, compared to the previous set of ten markers. Conditional analysis was performed including and excluding the PAGE Immunochip dataset, to evaluate the impact of only using cohorts that have genome-wide coverage. Excluding PAGE, our stopping criterion was achieved after 170 markers had been identified through conditional analysis, whereas including PAGE it was achieved after 200 markers. To ensure a fair comparison, we continued to run conditional analysis excluding PAGE until 200 markers had also been identified. In each case, the MHC contains the most markers identified through feature selection (18 with PAGE and 5 without it), reflecting its key role in the genetic signature for psoriasis subtypes^13,14^. Nevertheless, 91% of markers identified (through conditional analysis) with PAGE and 98% identified without PAGE were outside the MHC region. Our results illustrate that loci outside the genome-wide significant region can still play important roles in psoriasis subtype classifications (Supplementary Table 2, Supplementary Data 1). Only 15 of the 200 markers selected through conditional analysis without the PAGE cohort were also well-imputed (*r*^2^ ≥ 0.7) in PAGE. In addition, indicating that genotype imputation is key to integrating different datasets in our machine-learning pipeline, none of the markers selected through conditional analysis were genotyped across all cohorts; only 11.5% (23 out of 200) of markers selected including PAGE and 6.5% (13 out of 200) of markers selected excluding PAGE, respectively, were genotyped in at least one cohort. To further improve the robustness of our models when applied to new data, we also implemented an ensemble-based approach, whereby conditional analysis is repeated multiple times on different subsets of samples (see Discussion).

### Predicting psoriasis subtypes

**Fig. 4:**
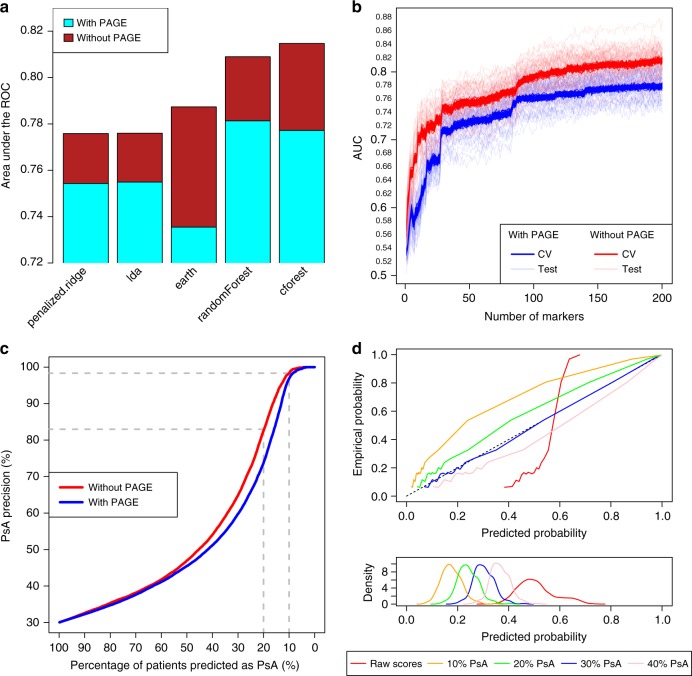
Risk prediction and assessment. **a** Benchmarking performance, on the cross-validation set, of the top five classifiers (*penalized.ridge* logistic ridge regression; *lda* linear discriminant analysis; *earth* multivariate adaptive regression splines; *randomForest* random forest; *cforest* conditional inference forest) out of 26 MLR classifiers in the complete benchmark (Supplementary Figure 4). **b** Classifier performance (on both the CV and test set), calculated using the area under the receiver operator curve (AUC). **c** Trade-off between precision and recall when predicting different proportions of samples as having PsA in hold-out test set. **d** Evaluation of classifier calibration, under different prior probabilities for PsA (on the 3:7 PsA/PsC ratio test set), which we subsequently used to predict the risk of patients with, as of yet, undiagnosed psoriasis subtype developing PsA

We compared the performance of a wide range of machine-learning classifiers using cross-validation (CV) through the MLR^32^ package in R (Fig. 4a, Supplementary Figure 7); 70% of the 26 classifiers we evaluated had an AUROC > 0.7, indicating the features we selected are robust in classifying psoriasis subtypes. To minimize the impact of any random noise, we repeated the results over 50 CV trials and calculated the mean AUROC (Supplementary Figure 8). When using all cohorts, Random Forest, an ensemble learning approach, achieved the highest mean AUROC (0.78). However, when excluding the PAGE cohort in the model, another ensemble learning approach, conditional inference forest, achieved the highest mean AUROC (0.82). The main difference between these two approaches is that, in Random Forest, individual trees are constructed using Gini impurity (a measure of how well separated the PsA/PsC classes are), whereas in conditional inference forest, trees are constructed according to a permutation test (to compare the correlation of the class variable with each of the predictors, i.e., genetic markers). Interestingly, classifier performance was consistently enhanced by 5% when PAGE was removed, when using the best classifier in each case. This difference was confirmed using a separate hold-out test set (which also gave 0.78 and 0.82 mean AUROCs with and without PAGE, respectively) (Fig. 4b). The AUROC for the test set also increases with the number of samples in the training set (Supplementary Figure 9). Using the ensemble-based approach for conditional analysis and model training, we found shrinkage discriminant analysis to be the most effective classifier, with an AUROC of 0.82 (Supplementary Figure 10). While AUROC is often used to measure classification performance, it is not sensitive to class (i.e., PsA/PsC subtype) proportions, and might not have sufficient translational impact, especially when the prevalence of the disease of interest is low or if the prevalence of the disease subtype is different from the proportion in the training dataset. We therefore evaluated additional metrics of classifier performance (i.e., precision, specificity and recall; Fig. 4c), and used an independent test set (10% of the samples selected at random and held out until after classifier selection and tuning was completed) with 3:7 ratio of PsA and PsC samples, assuming 30% of PsA prevalence among psoriatic patient^2^. We achieved over 90% precision (Supplementary Table 3) on average for the top 5% of patients predicted to have PsA (with 100% specificity and 16% recall). There is a trade-off between precision and recall, as for example when conditional analysis was not performed in each fold of the ensemble (i.e., our original approach), predicting the top 10% of patients to have PsA provides a recall of 33%, but extending our prediction to the top 20% increases recall to 55%. We also evaluated the performance when MHC variants were used on their own to provide PsA vs. PsC risk assessment. When restricting the model trained without PAGE to its 5 MHC markers (Supplementary Data 1), the AUROC was reduced to 0.58 in cross-validation and 0.54 on the training dataset. These results suggest that, whilst the MHC is the only genome-wide significant locus comparing PsA and PsC, using it alone is not the most effective approach.

For comparison, we also created classification models for distinguishing PsA, PsC and PsV samples from controls. Using a comparable and more time-effective approach, elastic net regression, the AUROC for PsA vs. Control was 0.91 in cross-validation and 0.92 on the test set; for PsC vs. Control, the AUROC was 0.88 in cross-validation and 0.89 on the test set; and for PsV vs. Control, the AUROC was 0.89 in both cross-validation and testing.

### PsA-risk assessment

We implemented an approach to assess the risk psoriasis patients will later develop PsA, by incorporating prior PsA prevalence (among psoriasis patients) using Bayes’ theorem. We evaluated the risk for each patient with 10, 20, 30 and 40% prior prevalence for PsA, and calibrated the classifier scores produced during cross-validation (using conditional inference forest without PAGE), then applied this to the (3:7 PsA/PsC ratio) independent test set (Fig. 4d). As expected, a (correct) prior of 30% resulted in the most accurate risk assessment. If we underestimated the PsA prevalence (e.g., 10%), then we would also under-estimate the posterior probability for patients to develop PsA. In addition, the calibrated probability predictions (regardless of prior probability) followed the empirical PsA prevalence (calculated from the actual PsA/PsC labels) much more closely than the raw classifier scores (Fig. 4d). This illustrates the classifier calibration approach is superior to the use of raw classifier scores, which are based on the prevalence in the training set. Sensitivity analyses using other prior prevalence also provided better results than using raw classifier scores, indicating the adjustment of subtype prevalence in the risk assessment is important. We then applied our classifier calibration approach to assess the PsA risk among 2471 psoriatic patients with undiagnosed subphenotype status and genome-wide coverage; interestingly, we identified 17 patients with >80% of risk of developing PsA (including two patients with >98% risk). Our results illustrate genetic data can serve as part of a robust personalized healthcare metric to aid diagnosis of psoriasis subtypes.

## Discussion

In this work, we combined advanced machine-learning techniques with the largest number of genotyped PsA and PsC samples so far, to reveal a set of comprehensive genetic features (through statistical imputation with a combination of reference panels) and predict the risk of PsA. Our study illustrates nine new loci for psoriasis and psoriasis subtypes and suggests robust prediction of PsA and PsC can be achieved using genetic data alone.

We ensured the robustness of our study by performing extensive quality control, through relatedness testing and rephenotyping of samples. We also used cross-validation to train our classification model and held out a subset of samples for testing the fitted model. To evaluate the translational impact of our machine-learning approaches, we selected samples for the test set proportional to the prevalence of PsA (30%^2^) expected among psoriasis patients. However, we sampled the training set as an even proportion of psoriasis subtypes, to prevent over-fitting to any one class, and selected the samples for the training and test set at random, to avoid any systematic bias. Since markers were selected for the classification models using stepwise conditional meta-analysis on the entire dataset, there is a risk of selection bias in the classifier performance. To evaluate this, we reperformed direct PsA vs. PsC meta-analysis using only samples in the training set (to ensure the test set remained completely independent), and then selected markers with association *p* ≤ 0.05 to train an elastic net model. Comparable with our original approach (Fig. 4b), the AUROC was 0.81 in cross-validation and 0.77 on the test set, but the elastic net approach selected 2948 markers to achieve this. Restricting the model to the most informative 226 markers (*λ* = 0.0227) gave 0.80 AUROC in both cross-validation and testing, and the AUROC on the test set does not start to decrease until ~500 markers are included in the model (Supplementary Figure 11). This supports our decision to select 200 markers for our model, since the AUROC is maximized while minimizing the potential for over-fitting. As the markers identified by our approach are all common variants (MAF ≥ 0.01), probes can be designed to capture their genotypes, and 200 markers is a small enough number to allow a relatively inexpensive chip design. When assessing PsA risk, we calibrated the classifier scores by applying Bayes theorem to the cross-validation results (with various prior prevalence for PsA) and confirmed the accuracy of our probability predictions on the separate test set. All these steps are critical to avoid over-fitting and ensure the robustness of our machine-learning approaches.

To further evaluate the robustness of our approach, we left out each cohort one at a time and then trained on the remaining cohorts. The AUROC, testing on the left out cohorts, was 0.74 for CASP, 0.80 for Exomechip, 0.74 for Genizon and 0.55 for Kiel GWAS (we are unable to leave out PsAGWAS, as it does not contain any PsC samples). To address the lower than expected performance on certain left out cohorts, we also implemented an ensemble approach: we first set aside 10% of samples (with 3:7 PsA/PsC ratio) as a test set, then the remaining samples were divided into tenfolds (preserving the PsA/PsC ratio for each cohort); stepwise conditional meta-analysis was applied separately to each fold. and we created ten different models (one for each fold) before combining their predictions (on the common test set). The resulting 154 markers from each fold can be found in Supplementary Data 2. Instead of including all the markers in a single model (as with our original approach), we trained ten separate models using the data and markers from each fold, and then combined the predicted PsA samples according to the rank of their classification scores from each model. The ensemble approach achieved the same 0.82 AUROC (in cross-validation and testing) as our original technique (Supplementary Figure 12), with a slight reduction in precision (Supplementary Figure 13) recall and specificity (Supplementary Figure 14). However, our ensemble approach achieved a higher AUROC on each of the left out cohorts: 0.75 on CASP, 0.84 on Exomechip, 0.96 on Genizon, and 0.86 on Kiel, suggesting this approach to classification may be more robust when generalizing to new samples.

The new locus identified by our study (rs806349) is located inside an intron of *DLEU1*, a gene that plays a role in the regulation of apoptosis^33^. This suggests a way the locus may contribute to psoriasis, since keratinocytes from psoriatic skin have been found to be resistant to apoptosis and increase in apoptosis is associated with healing after photochemotherapy (PUVA)^34^. Although in knockdown experiments with mice^33^, *DLEU1* was previously shown to affect the expression of apoptotic genes such as *BCL2* and *BAX,* which have been found to be differentially expressed in psoriasis^35^, the *DLEU1* locus is a new genome-wide significant finding for psoriasis. It is interesting that this locus is also known to be associated with primary biliary cirrhosis^28^ and multiple sclerosis^29^. The most significant marker at this locus in our meta-analysis was considered a likely candidate to be a causal variant in multiple sclerosis, due to its proximity to transcription factors binding sites^29^. In fact, psoriasis shares many susceptibility regions with various autoimmune diseases^20^. This points to an ancillary benefit of our pipeline: by identifying patients with an elevated risk of PsA early (i.e., before symptoms appear), we not only improve personalized healthcare for psoriasis patients, but our pipeline can also be extended to differentiate comorbidity rates for psoriasis subtypes, such as cardiovascular disease, metabolic syndrome, and inflammatory bowel disease (IBD)^36^. All the new loci we identified for psoriasis subtypes (PsA and PsC) are already known loci for PsV. In addition, no loci outside of the MHC were genome-wide significant in direct or indirect PsA vs. PsC meta-analysis. Nevertheless, by combining multiple markers that have not yet achieved genome-wide significance, we are able to robustly distinguish between PsA and PsC subtypes. This reaffirms that, although the genetic differences between PsA and PsC are subtle, sufficient useful information exists in the genetic data to be taken advantage of by advanced machine-learning techniques and used as the basis for a clinically validated risk metric.

Machine-learning approaches have been applied to other types of -omics data, to study or classify psoriasis: random forests have been used to predict psoriasis from transcriptome data^37^ and electronic records^38^; support vector machines have been used to predict psoriasis from dermoscopy images^39^. Here, we applied machine-learning toward the production of a metric for predicting the risk of psoriasis subtypes among psoriasis patients, using purely genetic data. With regards to other autoimmune diseases, classification has been used to distinguish subtypes of IBD^23^ using genetic markers, with comparable accuracy (AUROCs of 0.86 and 0.83 for Crohn’s disease and ulcerative colitis, respectively). However, we have attempted to make our work translationally relevant, by combining PsA and PsC prediction into a single model, which can produce calibrated risk predictions that have been tested against realistic prevalence of PsA. In addition, we can achieve good performance using fewer genetic markers (our machine-learning approaches used the same 200 markers for PsA and PsC, compared to the separate sets of 573 markers for Crohn’s disease and 366 markers for ulcerative colitis in the IBD paper^23^). We used the default parameters for each classification algorithm (Supplementary Table 4), to make it easier for other researchers to reproduce our results. However, a caveat in the application of our pipeline to new cohorts or other diseases is that clinical, demographic or genotyping differences may make it difficult to train a model on one cohort and apply it to another. In order for the model to learn the cohort-specific parameters for optimized performance, the effects of these markers can be first modeled in the cohort before applying for future subtype risk assessment, thus ensuring the specific properties of their cohort are addressed. A potential limitation of using conditional analysis for marker selection (particularly when the sample size is small, such as in each fold of our ensemble approach) is that, as more markers are added to the conditional analysis, the separation of variables will eventually prevent the identification of the next marker on which to condition. This limits the total number of markers that can be selected for classification (by conditional analysis). Furthermore, we should point out that the machine-learning techniques employed in our study (random forest/conditional inference forest, shrinkage discriminant analysis, and elastic net) use different numbers of markers, and this is important to consider when comparing their performance.

Personalized approaches to healthcare have the potential to improve PsA prediction, management, and treatment by identifying subpopulations of patients for which individualized healthcare plans can be provided^40^. Instead of treating all patients suffering from psoriasis/PsA in the same way, personalized medicine can significantly improve the efficacy and efficiency of healthcare, by providing customized disease management through translational research^41^. For example, more than 30% of PsA patients do not respond sufficiently to TNF-α blockers^42^. Our pipeline could be used to develop individualized therapies that identify genetic signatures to differentiate PsA treatment responses^43^, thus limiting the use of ineffective and unnecessary treatments, though heritability of treatment response can play a major role, as a previous attempt using genetic data to predict patients’ response to anti-TNF drugs in rheumatoid arthritis failed to improve predictive performance compared to clinical traits^44^. The value of our work (to provide an accurate risk metric for PsA) is therefore high, both in terms of economic costs and in better outcomes for the patients. The risk assessment model we have developed has the potential to serve as a PsA signature in dermatology clinics and identify patients with psoriasis who are likely to develop PsA. This would advance clinical practices by reforming disease screening, prognosis, and treatment options including enhancing the design of clinical trials to determine whether PsA can be delayed or prevented with more aggressive treatment for certain individuals.

Early PsA diagnosis is essential^5,6^ for improving quality of life and reducing the economic burden to society. The pipeline we have developed represents a systematic strategy for quantitative risk assessment, before symptoms (joint pain, inflammation and damage) appear. We have identified new loci, shown that the genetic differences between PsA and PsC are due to regulatory elements, developed a robust metric for distinguishing the two subtypes and provided a framework for expansion with other kinds of data including transcriptomic and proteomic data, which are likely to become widely available in the coming era of personalized medicine.

## Methods

### Data processing

We defined the PsC subtype as PsV patients who, at the latest evaluation, have had psoriasis symptoms for over 10 years, without being diagnosed with PsA. All PsV patients were diagnosed by dermatologists and the PsA status was evaluated by rheumatologists (and/or dermatologists with specialized training in the diagnosis of PsA). Samples from each cohort were quality controlled as described previously^13,20^, and relatedness testing was performed to ensure only independent samples were used (we removed one of the duplicates or first/second degree relatives). All the samples in our study are of Caucasian descent and samples were excluded if they had substantial non-European admixture. X chromosome genotypes were used to validate gender. Population stratification was addressed using principal components analysis and geographic indicator covariates. Furthermore, markers with <0.01 MAF (minor allele frequency), <95% genotype call rate, <1 × 10^−6^ Hardy–Weinberg *p* value were removed. All human subjects provided written informed consent and were enrolled according to the protocols approved by the institutional review board for human subject research of each institution, in adherence with the Declaration of Helsinki principles.

### Phasing and imputation

Phasing was determined using ShapeIT^45^, to improve the accuracy and speed of imputation through efficient graph-based calculations for statistical haplotype estimation that scale linearly with the number of samples and markers. Imputation was performed for SNPs and INDELs using Minimac3^46^. We retained only markers that are well-imputed (imputation quality *r*^2^ ≥ 0.7) in at least one cohort for at least one reference panel; when the same marker was well-imputed in both the 1KG and HRC panels, we used the imputed dosage from the panel with the higher imputation quality. HLA markers were imputed using the Type I Diabetes Genetics Consortium (T1DGC) reference panel^47^. We modified the imputation tool (SNP2HLA) to take advantage of the increased accuracy of imputation in Beagle 4.1^48^.

### Association analysis

We used the logistic regression (Wald) test in the latest version of PLINK (2.0)^49^, which has implemented a more efficient statistical algorithm through bit manipulation and parallelism^50^. Variants with <1% minor allele frequency were excluded. In addition, top principal components and geographic cohort indicators were included as covariates^13,20^.

### Meta-analysis

We performed meta-analysis, using the inverse variance approach implemented in METAL^51^, to combine effect sizes and standard errors across the six cohorts. Genomic inflation factors were also used to control population stratification. Indirect meta-analysis was conducted by computing the statistic $\chi _{{\mathrm{PsA}}/{\mathrm{PsC}}}^2 = \frac{{\left( {\beta _{{\mathrm{PsA}}} - \beta _{{\mathrm{PsC}}}} \right)^2}}{{V_{{\mathrm{PsA}}} + V_{{\mathrm{PsC}}} - 2\rho _{{\mathrm{PsA}},{\mathrm{PsC}}}\sqrt {V_{{\mathrm{PsA}}}V_{{\mathrm{PsC}}}} }}$, where *β*_PsA_ and *β*_PsC_ are the log odds ratios for PsA vs. Control and PsC vs. Control meta-analyses, respectively; *V*_PsA_ and *V*_PsC_ are the corresponding variances, and *ρ*_PsA,PsC_ is the correlation between the two log odds ratios. Since there is no analytic approximation for the correlation between odds ratios, we assumed it is zero (deliberately making the test more conservative^13^). The test statistic follows a chi-squared distribution (with one degree of freedom), so we retrieved the resulting *p* values from the corresponding cumulative distribution function.

### Machine-learning for subtype prediction and risk assessment

This was achieved by calculating the mean area under the receiver operator curve (AUROC) in tenfold cross-validation, using 90% of the samples (randomly selected) for training; 10% of the samples were held out (as a test set) until after classifier selection and tuning was completed (Fig. 1b). In addition to AUROC, we used precision (the proportion of subtype prediction that was accurate) and recall (the proportion of PsA/PsC patients successfully predicted to have PsA/PsC status, respectively) to evaluate the performance of the resulting classification model at distinguishing between psoriasis subtypes. We also estimated each individual’s probability of developing PsA, using Bayes’ theorem to integrate the prior prevalence of PsA, given a classifier score of ***s***: $P\left( {{\mathrm{PsA|}}s} \right) = \frac{{d(s|{\mathrm{PsA}})P\left( {{\mathrm{PsA}}} \right)}}{{d(s|{\mathrm{PsA}})P\left( {{\mathrm{PsA}}} \right) + d(s|{\mathrm{PsC}})P\left( {{\mathrm{PsC}}} \right)}}$, where *P*(PsA) denotes the proportion of PsA among psoriasis patients, *P*(PsA) + *P*(PsC) = 1; and *d*(*s*|PsA), *d*(*s*|PsC) are kernel density estimates from the classifier scores for patients with known PsA and PsC status. We compared the effect of using different prior probabilities for *P*(PsA) and applied our model to assess the risk of PsA among patients who have shown psoriasis symptoms, but as of yet have unknown PsA/PsC status.

The default parameters for each classifier were used throughout our study^32^. We used the *p* ≤ 0.05 threshold in the elastic net model for PsA vs. PsC, but for other comparisons (PsA/PsC/PsV vs. Control), we adopted a slightly more stringent *p* ≤ 0.01 threshold for these models due to memory issue. Even so, we had to modify the code of R’s glmnet package slightly to allow classification on the large datasets on which we applied Elastic Net Regression, by replacing standard R calls to Fortran code with the dotCall64 library. For the conditional analysis stopping point (i.e., no substantial increase in the mean AUROC of ten consecutive markers), we evaluated the AUROC for all possible stopping points up until this criterion had been met (Fig. 4d).

### Code availability

In addition to the software packages previously described, some custom scripts were also used to produce the results. These may be found on GitHub (https://github.com/cutaneousBioinf).

## Electronic supplementary material

Supplementary Information

Description of Additional Supplementary Files

Supplementary Data 1

Supplementary Data 2

## Data Availability

Data from the CASP (phs000019.v1.p1), Exomechip (phs001306.v1.p1) and PsAGWAS (phs000982.v1.p1) cohorts are available in dbGap. The data for other cohorts are available upon request.

## References

[1] Alamanos Y, Voulgari PV, Drosos AA (2008). Incidence and prevalence of psoriatic arthritis: a systematic review. J. Rheumatol..

[2] Mease PJ (2013). Prevalence of rheumatologist-diagnosed psoriatic arthritis in patients with psoriasis in European/North American dermatology clinics. J. Am. Acad. Dermatol..

[3] Ogdie A (2017). The preclinical phase of PsA: a challenge for the epidemiologist. Ann. Rheum. Dis..

[4] Helliwell PS, Ruderman EM (2015). Natural history, prognosis, and socioeconomic aspects of psoriatic arthritis. Rheum. Dis. Clin. N. Am..

[5] Gladman D. D., Thavaneswaran A., Chandran V., Cook R. J. (2011). Do patients with psoriatic arthritis who present early fare better than those presenting later in the disease?. Annals of the Rheumatic Diseases.

[6] Haroon Muhammad, Gallagher Phil, FitzGerald Oliver (2014). Diagnostic delay of more than 6 months contributes to poor radiographic and functional outcome in psoriatic arthritis. Annals of the Rheumatic Diseases.

[7] Ritchlin CT, Colbert RA, Gladman DD (2017). Psoriatic arthritis. N. Engl. J. Med..

[8] Villani AP (2015). Prevalence of undiagnosed psoriatic arthritis among psoriasis patients: systematic review and meta-analysis. J. Am. Acad. Dermatol..

[9] Coates LC, Hodgson R, Conaghan PG, Freeston JE (2012). MRI and ultrasonography for diagnosis and monitoring of psoriatic arthritis. Best Pract. Res. Clin. Rheumatol..

[10] Taylor W (2006). Classification criteria for psoriatic arthritis: development of new criteria from a large international study. Arthritis Rheumatol..

[11] Eder L (2014). Is the MAdrid Sonographic Enthesitis Index useful for differentiating psoriatic arthritis from psoriasis alone and healthy controls?. J. Rheumatol..

[12] Greb JE (2016). Psoriasis. Nat. Rev. Dis. Prim..

[13] Stuart PE (2015). Genome-wide association analysis of psoriatic arthritis and cutaneous psoriasis reveals differences in their genetic architecture. Am. J. Hum. Genet..

[14] Okada Y (2014). Fine mapping major histocompatibility complex associations in psoriasis and its clinical subtypes. Am. J. Hum. Genet..

[15] Winchester R (2012). HLA associations reveal genetic heterogeneity in psoriatic arthritis and in the psoriasis phenotype. Arthritis Rheumatol..

[16] Liu Y (2008). A genome-wide association study of psoriasis and psoriatic arthritis identifies new disease loci. PLoS Genet..

[17] Huffmeier U (2010). Common variants at TRAF3IP2 are associated with susceptibility to psoriatic arthritis and psoriasis. Nat. Genet..

[18] Ellinghaus E (2012). Genome-wide meta-analysis of psoriatic arthritis identifies susceptibility locus at REL. J. Investig. Dermatol..

[19] Nair RP (2009). Genome-wide scan reveals association of psoriasis with IL-23 and NF-kappaB pathways. Nat. Genet..

[20] Tsoi LC (2017). Large scale meta-analysis characterizes genetic architecture for common psoriasis-associated variants. Nat. Commun..

[21] Ellinghaus E (2010). Genome-wide association study identifies a psoriasis susceptibility locus at TRAF3IP2. Nat. Genet..

[22] Tsoi LC (2012). Identification of 15 new psoriasis susceptibility loci highlights the role of innate immunity. Nat. Genet..

[23] Wei Z (2013). Large sample size, wide variant spectrum, and advanced machine-learning technique boost risk prediction for inflammatory bowel disease. Am. J. Hum. Genet..

[24] McCarthy S (2016). A reference panel of 64,976 haplotypes for genotype imputation. Nat. Genet..

[25] The 1000 Genomes Project Consortium. A global reference for human genetic variation. *Nature* **526**, 68–74 (2015). 10.1038/nature15393 PMC4750478 26432245

[26] Gao X (2012). Genotype imputation for Latinos using the HapMap and 1000 Genomes Project reference panels. Front. Genet..

[27] Tsoi LC (2015). Enhanced meta-analysis and replication studies identify five new psoriasis susceptibility loci. Nat. Commun..

[28] Cordell HJ (2015). International genome-wide meta-analysis identifies new primary biliary cirrhosis risk loci and targetable pathogenic pathways. Nat. Commun..

[29] Andlauer TF (2016). Novel multiple sclerosis susceptibility loci implicated in epigenetic regulation. Sci. Adv..

[30] Lill CM (2015). Genome-wide significant association with seven novel multiple sclerosis risk loci. J. Med. Genet..

[31] Farh KK (2015). Genetic and epigenetic fine mapping of causal autoimmune disease variants. Nature.

[32] Bischl B (2016). mlr: machine-learning in R. J. Mach. Learn. Res..

[33] Lee S (2017). The effects of DLEU1 gene expression in Burkitt lymphoma (BL): potential mechanism of chemoimmunotherapy resistance in BL. Oncotarget.

[34] Laporte M, Galand P, Fokan D, de Graef C, Heenen M (2000). Apoptosis in established and healing psoriasis. Dermatology.

[35] Kocak M, Bozdogan O, Erkek E, Atasoy P, Birol A (2003). Examination of Bcl-2, Bcl-X and bax protein expression in psoriasis. Int. J. Dermatol..

[36] Husni ME (2015). Comorbidities in psoriatic arthritis. Rheum. Dis. Clin. N. Am..

[37] Ainali C (2012). Transcriptome classification reveals molecular subtypes in psoriasis. BMC Genom..

[38] Love TJ, Cai T, Karlson EW (2011). Validation of psoriatic arthritis diagnoses in electronic medical records using natural language processing. Semin. Arthritis Rheum..

[39] Shrivastava VK, Londhe ND, Sonawane RS, Suri JS (2015). Reliable and accurate psoriasis disease classification in dermatology images using comprehensive feature space in machine-learning paradigm. Expert Syst. Appl..

[40] Menter MA, Griffiths CEM (2015). Psoriasis: the future. Dermatol. Clin..

[41] Aronson SJ, Rehm HL (2015). Building the foundation for genomics in precision medicine. Nature.

[42] Huffmeier U, Mossner R (2014). Complex role of TNF variants in psoriatic arthritis and treatment response to anti-TNF therapy: evidence and concepts. J. Investig. Dermatol..

[43] Coates LC, FitzGerald O, Helliwell PS, Paul C (2016). Psoriasis, psoriatic arthritis, and rheumatoid arthritis: Is all inflammation the same?. Semin. Arthritis Rheum..

[44] Sieberts SK (2016). Crowdsourced assessment of common genetic contribution to predicting anti-TNF treatment response in rheumatoid arthritis. Nat. Commun..

[45] Delaneau O, Marchini J, Zagury JF (2012). A linear complexity phasing method for thousands of genomes. Nat. Methods.

[46] Das S (2016). Next-generation genotype imputation service and methods. Nat. Genet..

[47] Jia X (2013). Imputing amino acid polymorphisms in human leukocyte antigens. PLoS One.

[48] Browning BL, Browning SR (2016). Genotype imputation with millions of reference samples. Am. J. Hum. Genet..

[49] Chang, C. et al Second-generation PLINK: rising to the challenge of larger and richer datasets. *GigaScience* **4**, s13742-015-0047-8 (2015). 10.1186/s13742-015-0047-8 PMC4342193 25722852

[50] Hill, A. et al. Stepwise distributed open innovation contests for software development: acceleration of genome-wide association analysis. *GigaScience* 6, gix009 (2017). 10.1093/gigascience/gix009 PMC5467032 28327993

[51] Willer CJ, Li Y, Abecasis GR (2010). METAL: fast and efficient meta-analysis of genome-wide association scans. Bioinformatics.

