# Supplementary material for: Genetic signature to provide robust risk assessment of psoriatic arthritis development in psoriasis patients
Source: Nat Commun. 2018 Oct 9;9:4178. doi: 10.1038/s41467-018-06672-6 (PMC6177414; doi:10.1038/s41467-018-06672-6)
Supplement: Supplementary file 1 — Supplementary Information [file 41467_2018_6672_MOESM1_ESM.pdf]

Supplementary Figures

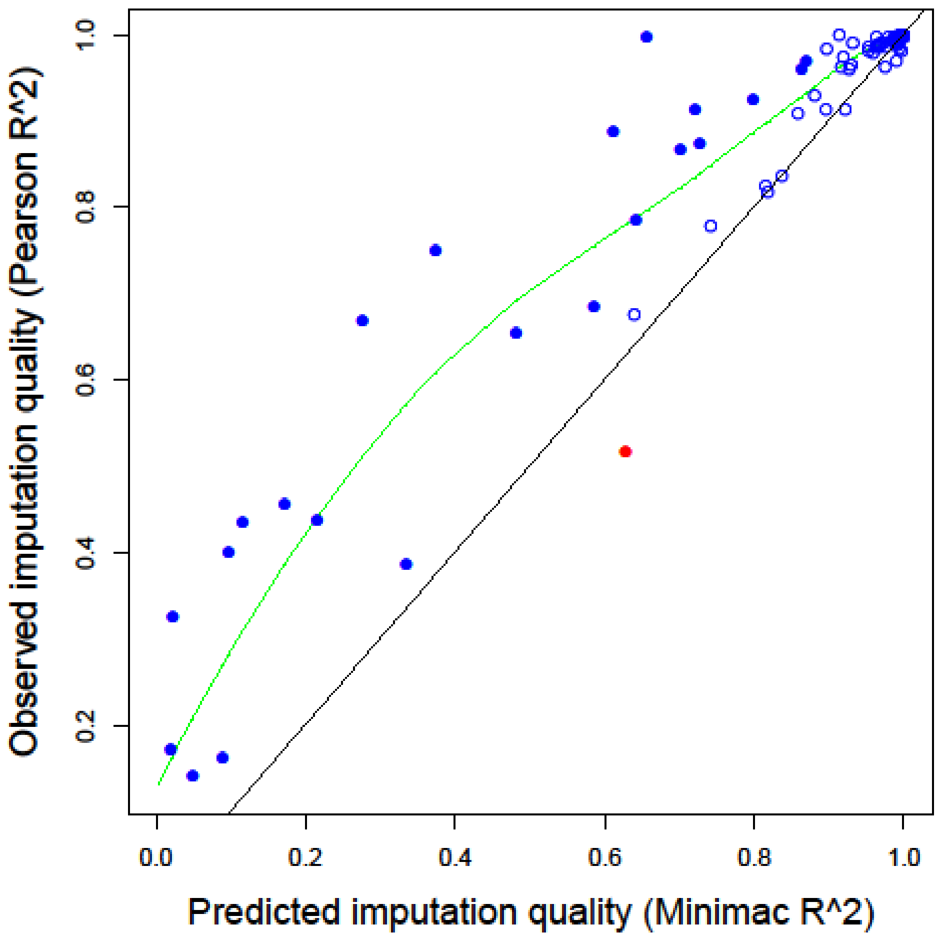

Supplementary Figure 1: Accuracy of genotype imputation. The predicted imputation quality from Minimac for the same 24 genotyped markers in CASP, PAGE and PsAGWAS was compared against the actual observed imputation quality measured by Pearson correlation coefficient with the genotyped samples. Filled blue dots indicate instances where the observed imputation quality was at least 10% higher than predicted, whereas red dots indicate instances where the observed imputation quality was at least 10% lower than predicted and hollow blue dots indicate a difference in imputation quality of less than 10%. The green line represents the robust LOESS curve for the data. In only one instance was the observed imputation quality substantially less than predicted, and in most cases the observed imputation quality was substantially higher. This suggests our genotype imputation was highly accurate, especially since we only include markers where the predicted imputation quality is greater than 0.7.

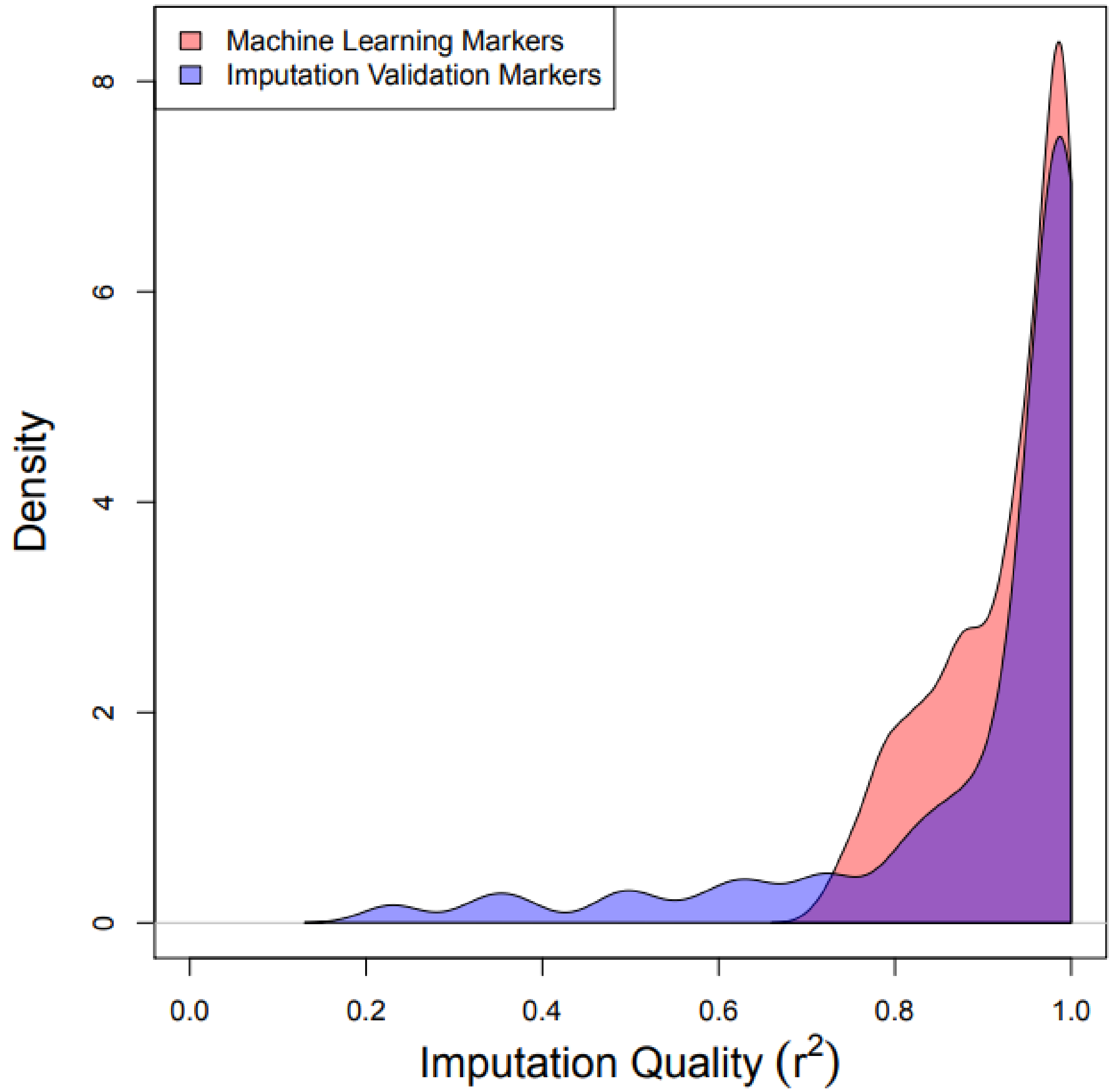

Supplementary Figure 2: Density Plot for Comparing Imputation Quality (with CASP, Exomechip and PsAGWAS). The distribution of predicted imputation quality for markers used to validate our imputation is shown in blue, whereas the distribution for the 200 markers used in our machine learning is shown in red.

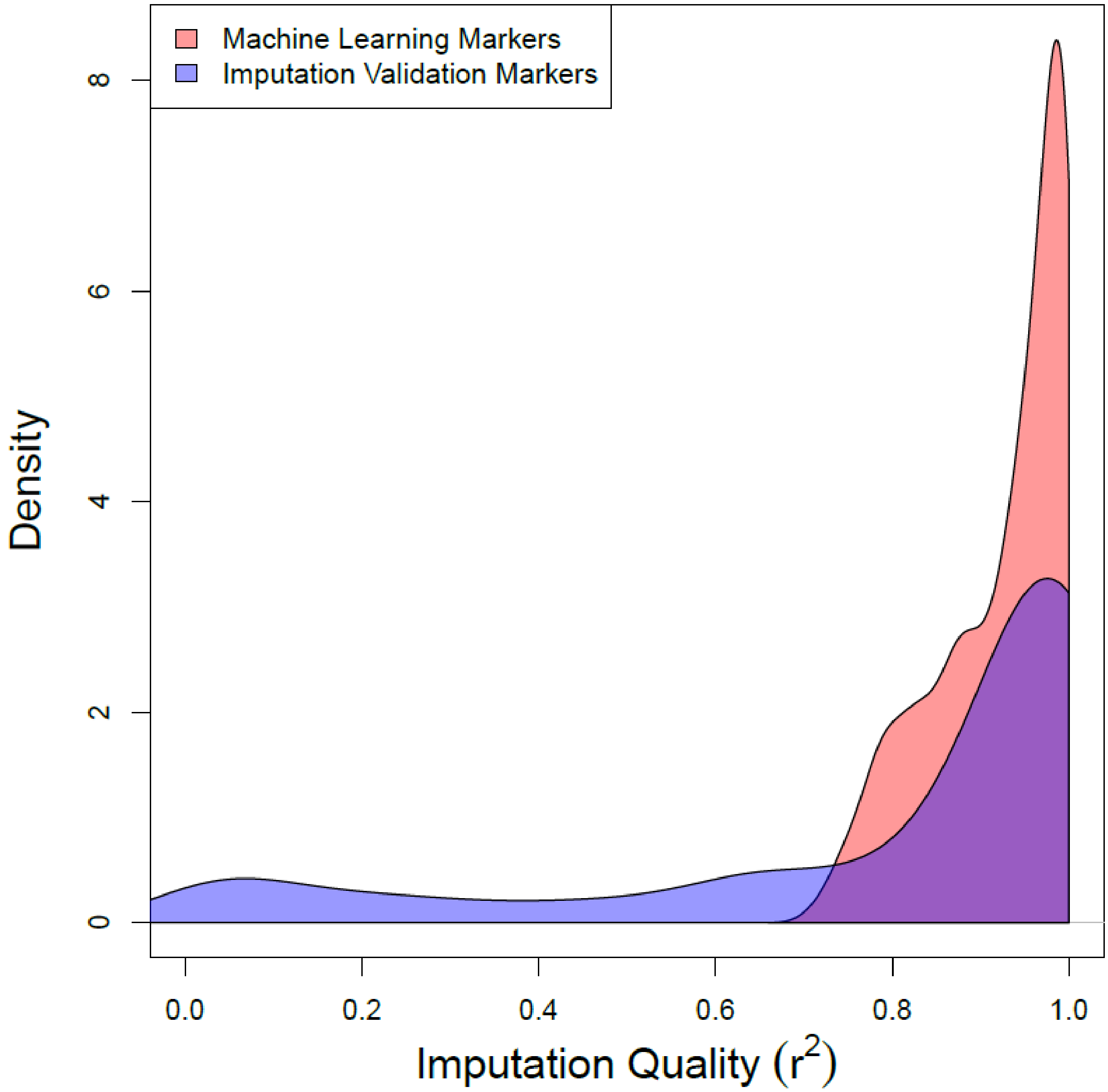

Supplementary Figure 3: Density Plot for Comparing Imputation Quality (with CASP, Exomechip, PsAGWAS and PAGE). The distribution of predicted imputation quality for markers used to validate our imputation is shown in blue, whereas the distribution for the 200 markers used in our machine learning is shown in red.

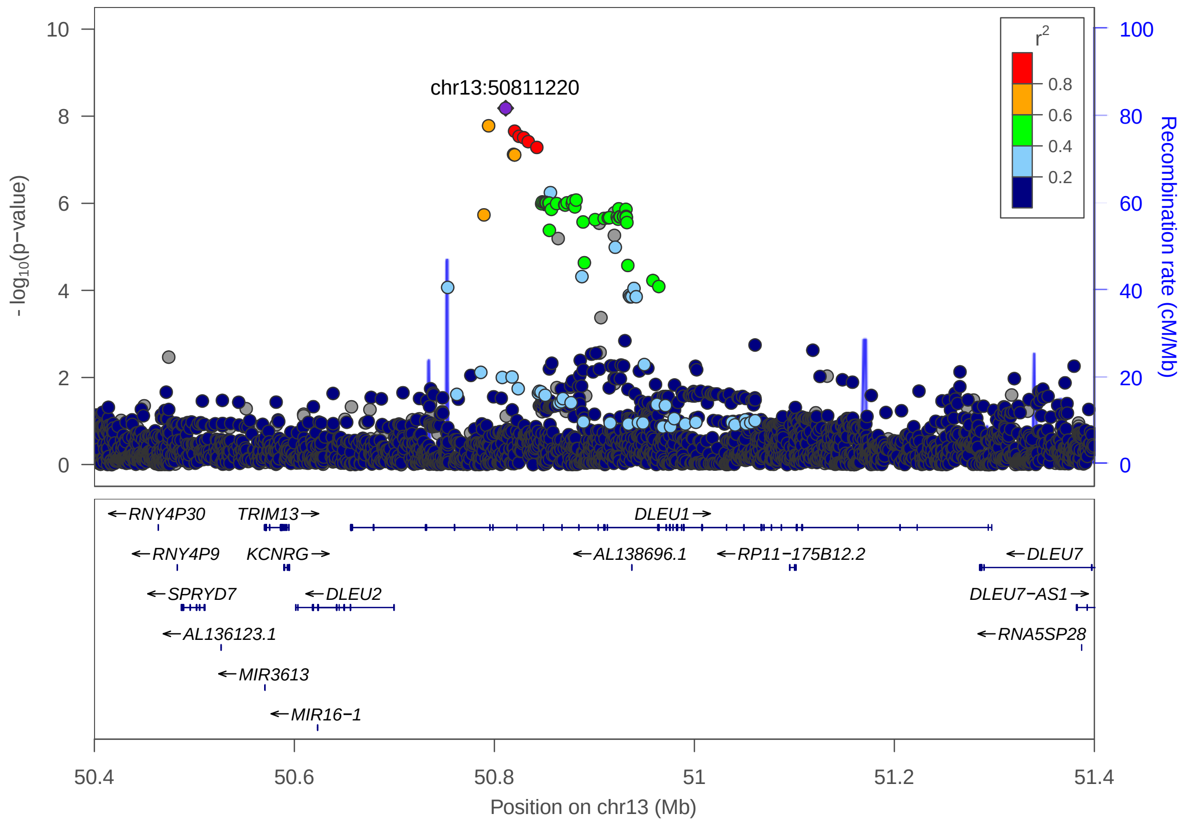

Supplementary Figure 4: LocusZoom plot for new PsV locus at 13q14.2 The plot shows that rs9591325 (chr13:50811220) lies inside an intron of *DLEU1*

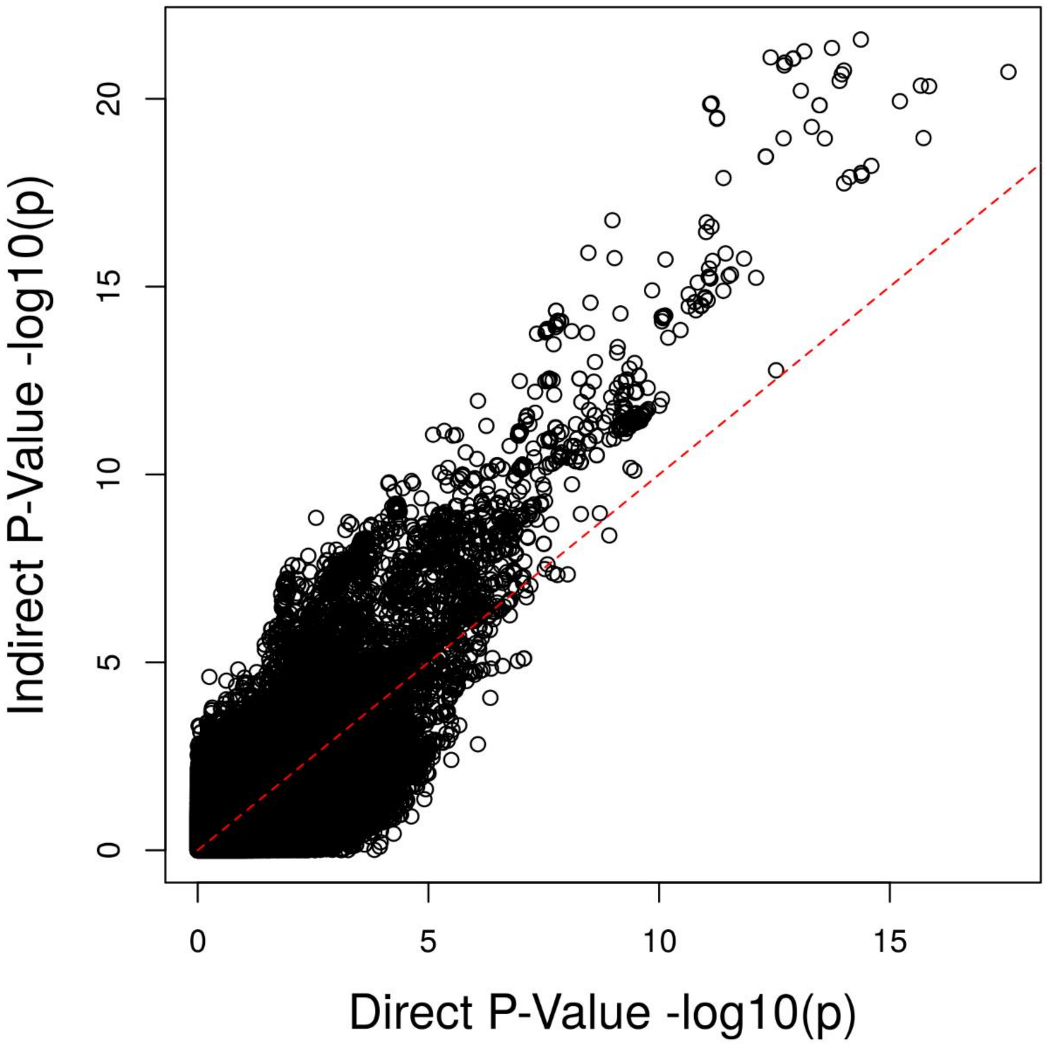

Supplementary Figure 5: Direct versus Indirect Meta-analysis We compared the p-values of almost 10 million genetic markers and found indirect meta-analysis (using all cohorts) has consistently more power to distinguish between PsA and PsC than direct meta-analysis (using all cohorts but PsA GWAS).

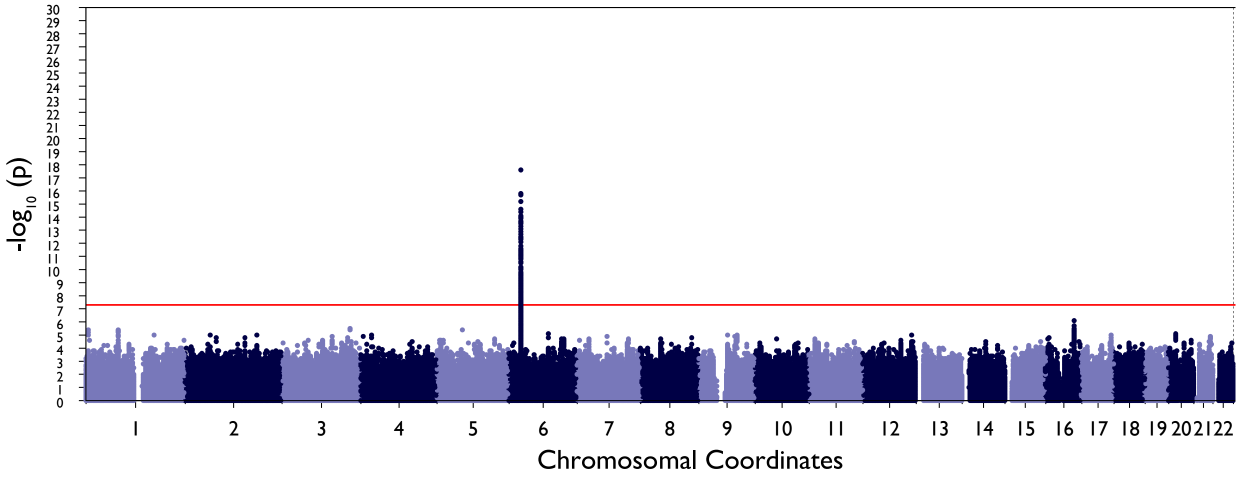

Supplementary Figure 6: Direct PsA versus PsC Meta-analysis In accordance with previous research, no genome-wide significant loci were identified outside of the MHC in chromosome 6

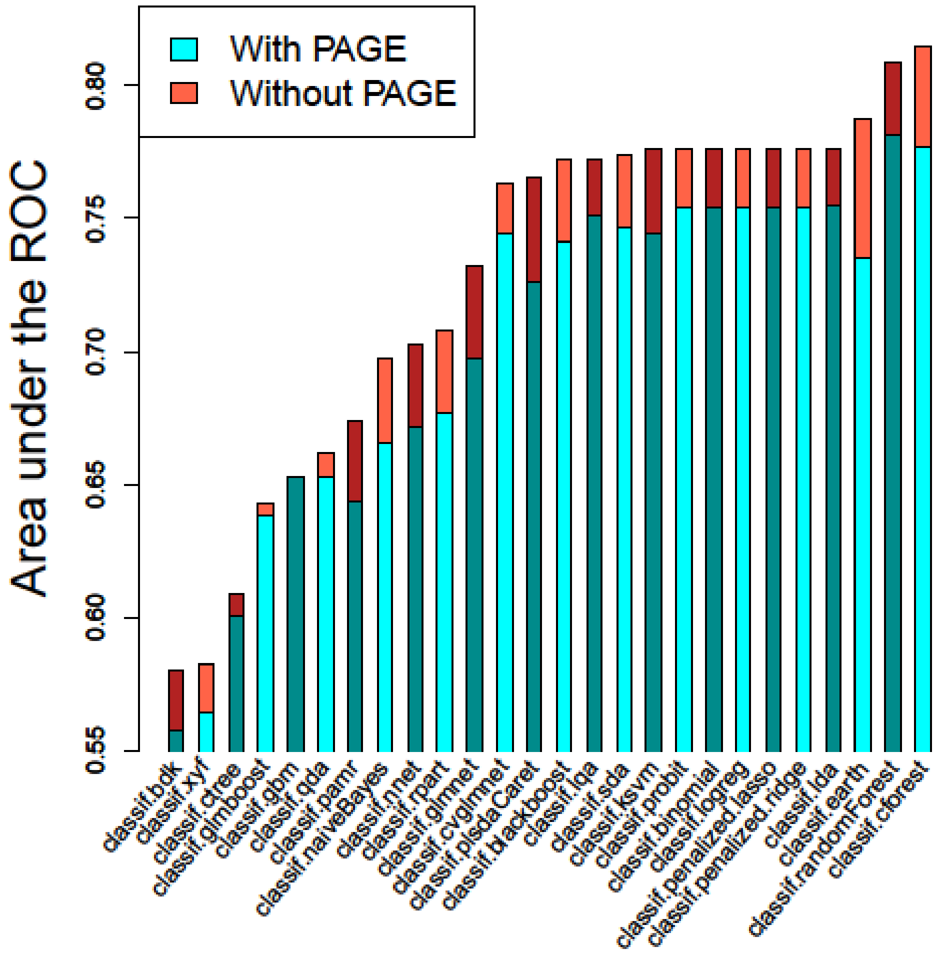

Supplementary Figure 7: Benchmarking the performance of 26 Classifiers in MLR in the cross-validation set for PsA vs. PsC prediction. For more details about each classifier see: http://mlr-org.github.io/mlr-tutorial/release/html/integrated_learners/

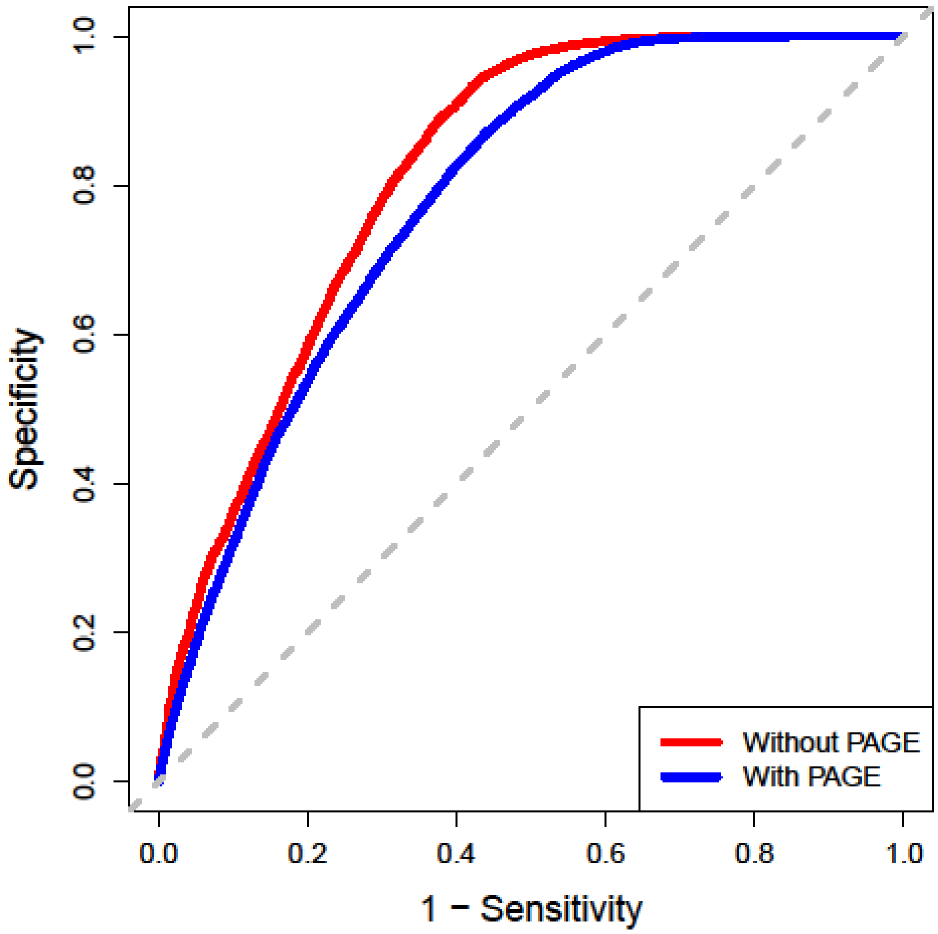

Supplementary Figure 8: Receiver Operator Curve (ROC). Comparing the specificity and sensitivity when classifying PsA vs. PsC with (Random Forest) or without PAGE (Conditional Inference Forest) averaged over 50 trials. The mean area under the ROC (AUROC) is 0.78 with PAGE and 0.82 without it.

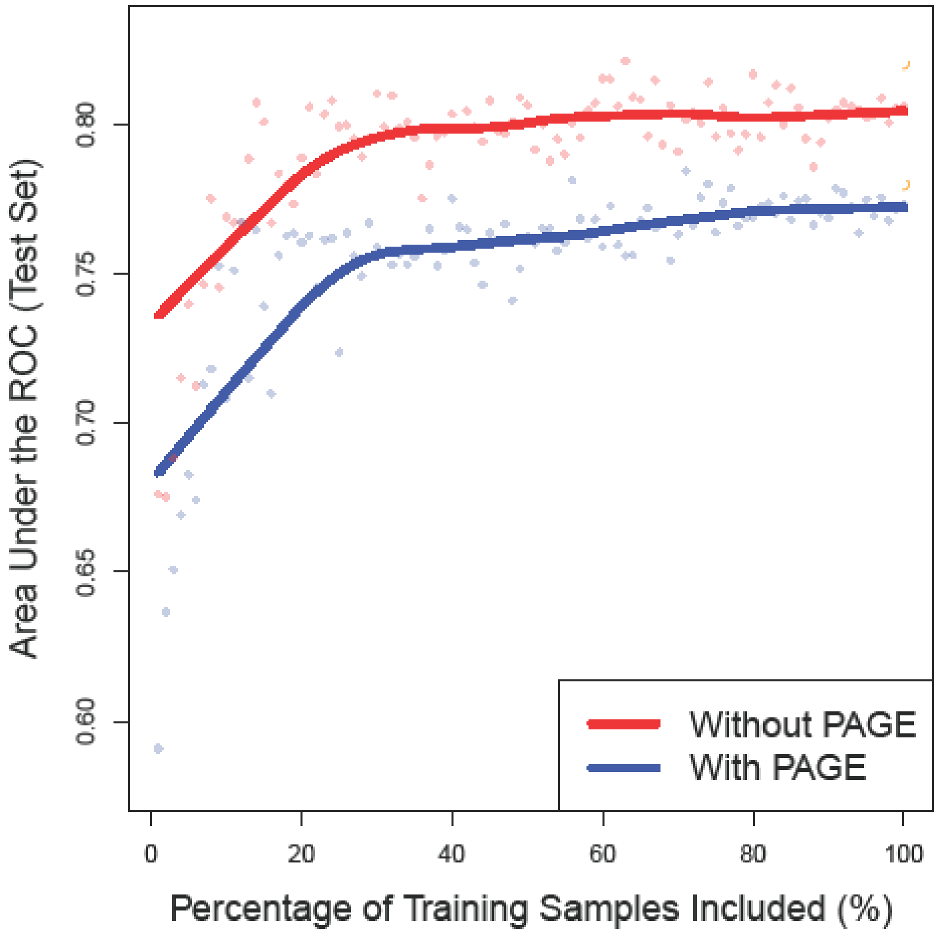

Supplementary Figure 9: Area under the ROC as a function of the sample size for PsA vs. PsC prediction. AUROC on the test set increases with the number of training samples, but the rate of increase slows down at around 30% inclusion. Classification with PAGE is performed using Random Forest, whereas classification without PAGE is performed using Conditional Inference Forest.

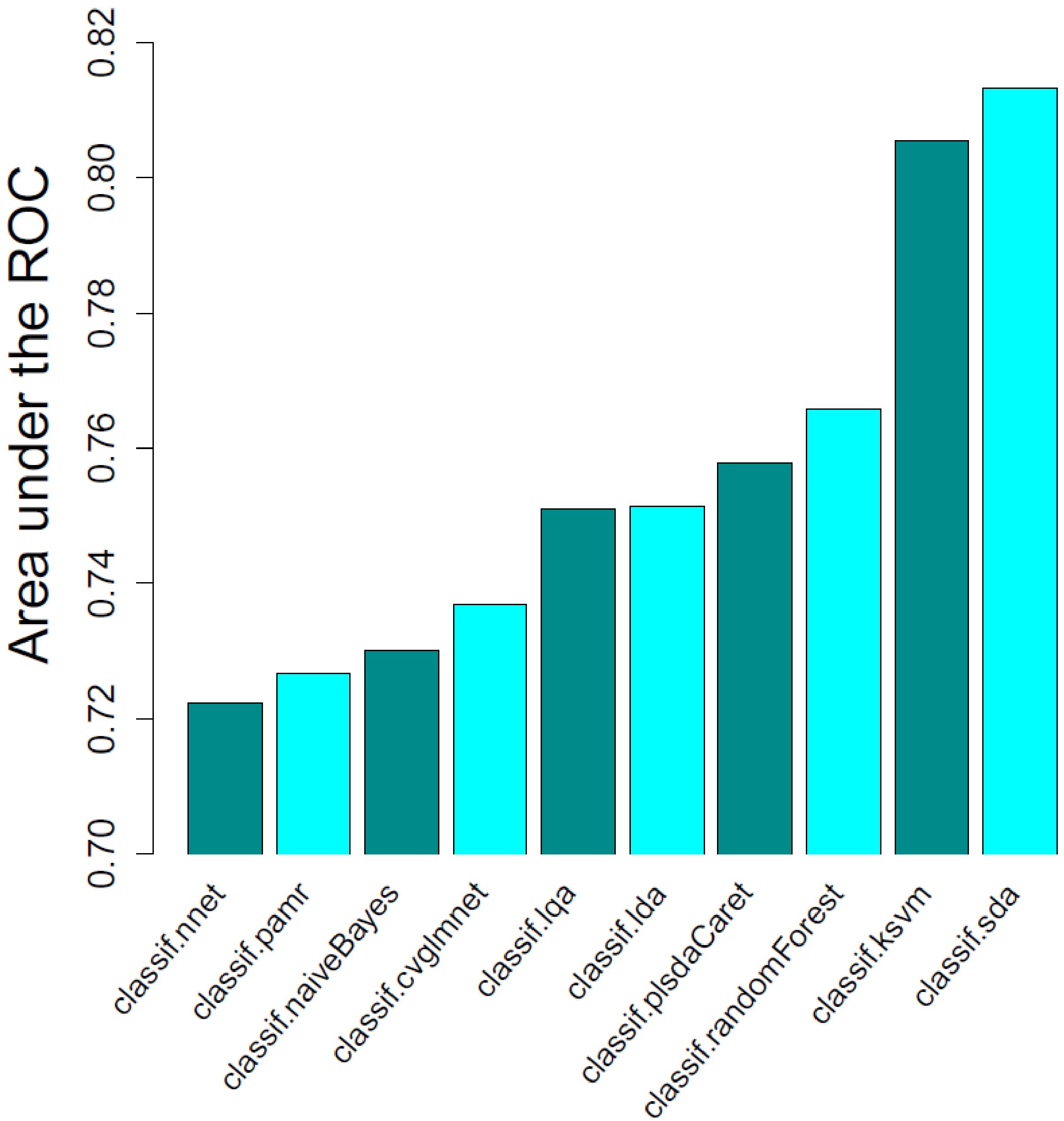

Supplementary Figure 10: Benchmarking the performance of the top 10 classifiers in MLR using our 10-fold ensemble approach. For more details about each classifier see: http://mlr-org.github.io/mlr-tutorial/release/html/integrated_learners/

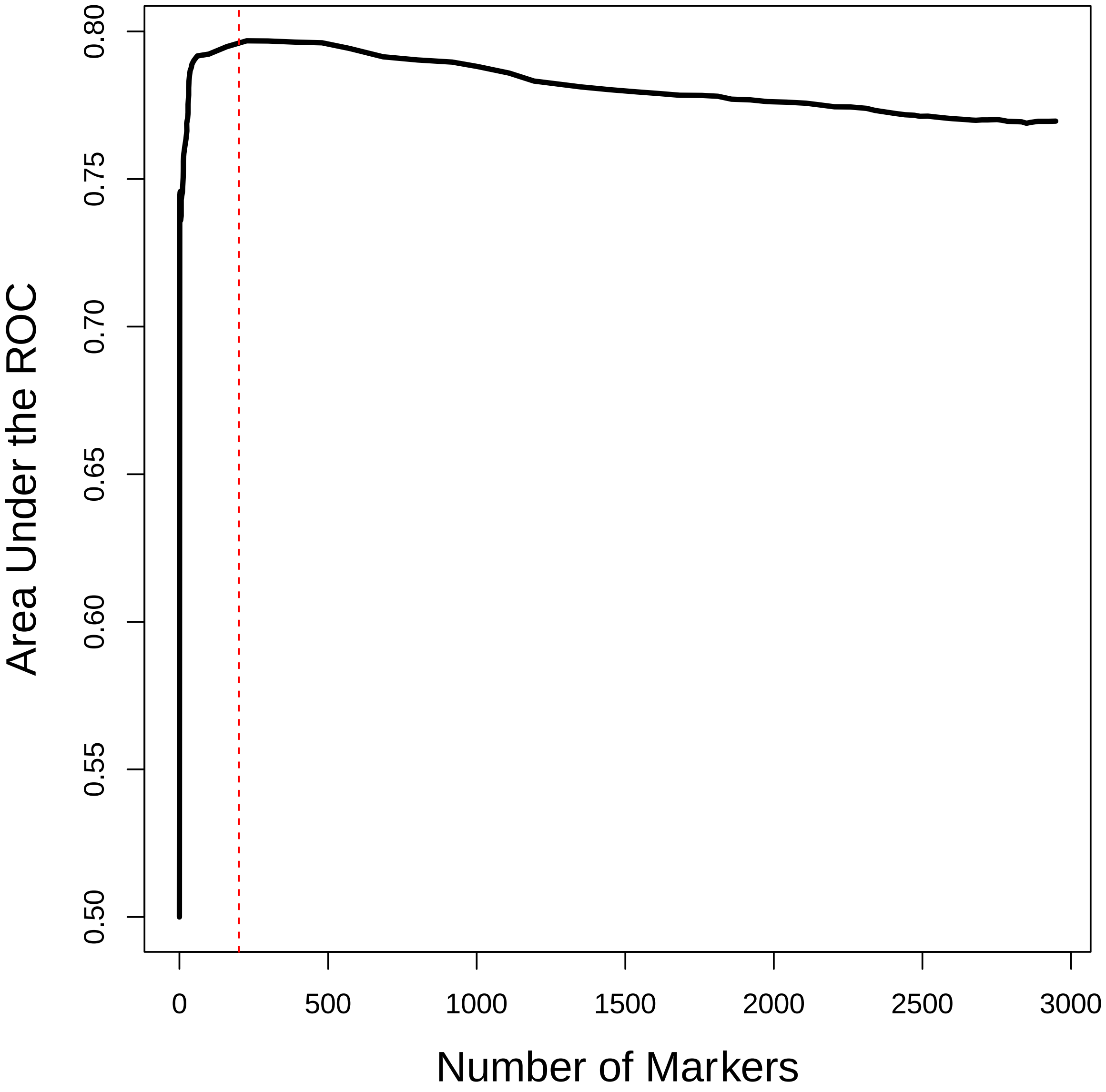

Supplementary Figure 11: Area under the ROC as a function of the number of markers included in the model for PsA vs. PsC prediction. AUROC on the test set increases with the number of markers until ~500 markers are included and then starts to decrease. By selecting 200 markers for use in our model, the AUROC is maximized, whilst minimizing the risk of over-fitting. The AUROC for this figure was calculated by training an elastic net on one of the randomly sampled training set, using markers that had p<=0.05 in direct PsA/PsC meta-analysis on the samples in the training set. We then plotted the AUROC produced when models with different numbers of markers were applied to the test set.

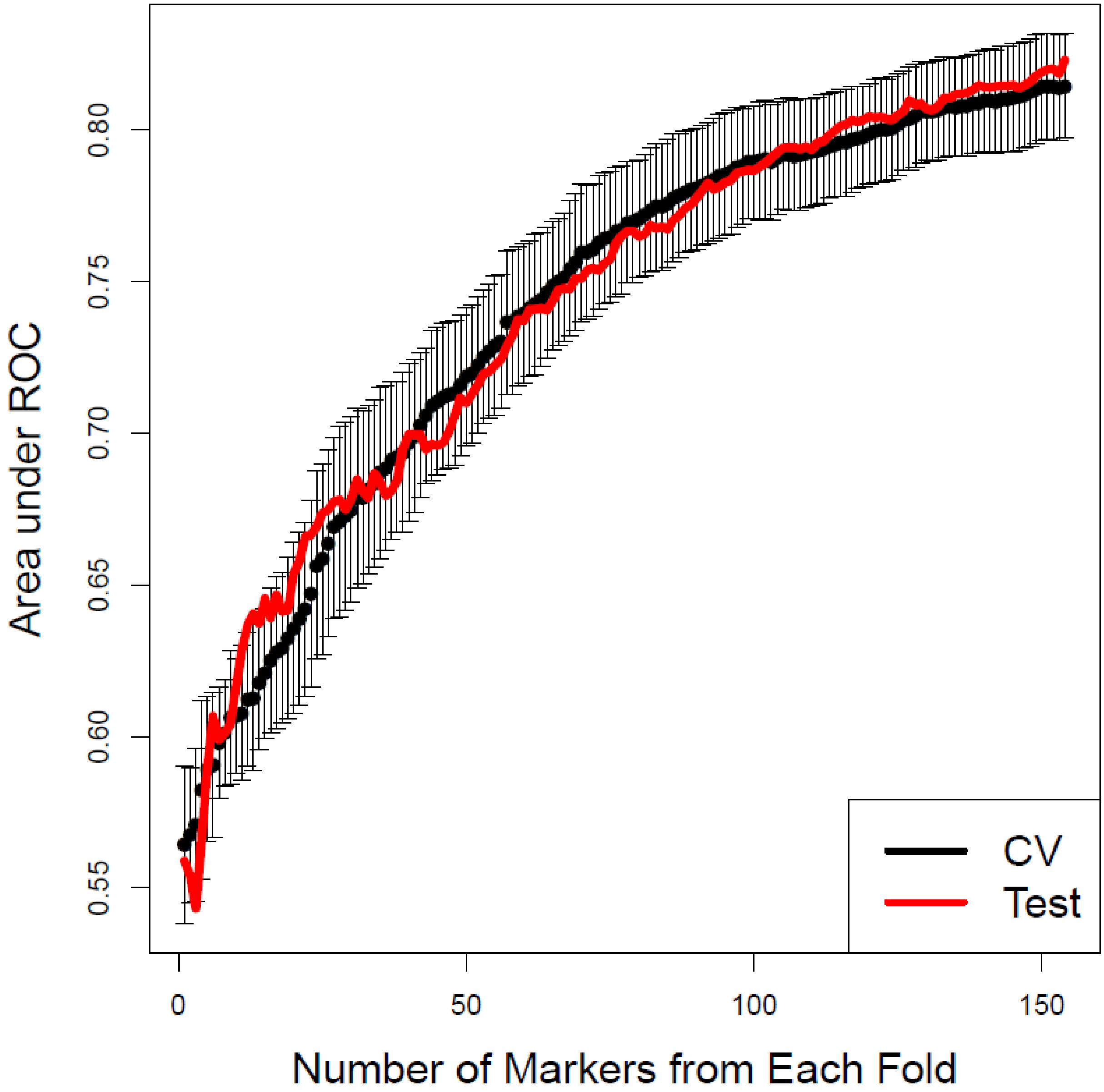

Supplementary Figure 12: Area under the ROC as a function of the number of markers in each fold of our 10-fold ensemble approach for PsA vs. PsC prediction. AUROC in cross validation and on the test set increases with the number of markers added. Error bars indicated plus or minus one standard deviation (±1SD) from the mean AUROC across the 10 folds (N=10).

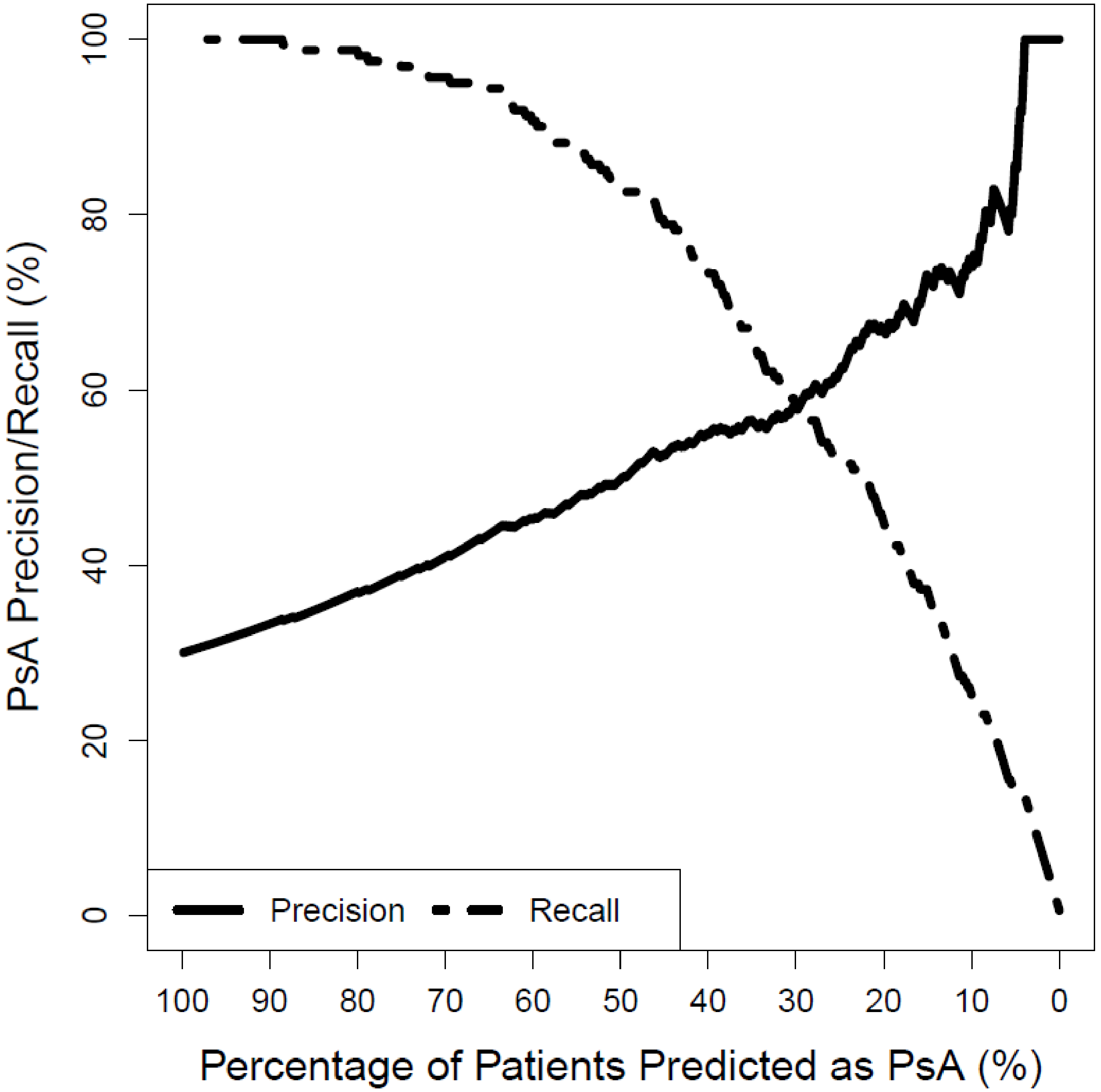

Supplementary Figure 13: Precision and recall as a function of the number of patients predicted to have PsA in each fold of our 10-fold ensemble approach for PsA vs. PsC prediction. Our approach achieves over 90% precision for a recall of around 16%.

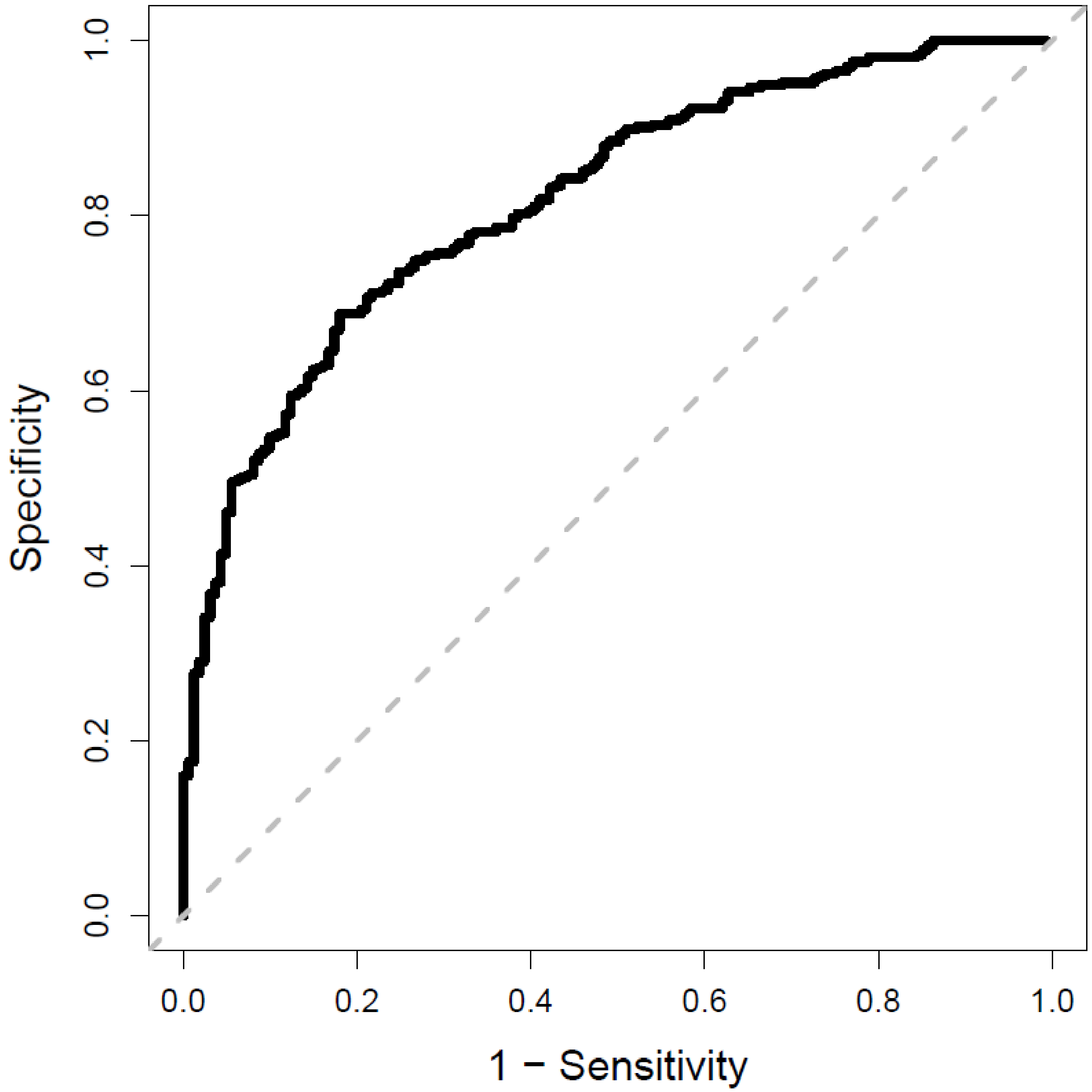

Supplementary Figure 14: Receiver Operator Curve (ROC). Comparing the specificity and sensitivity when classifying PsA vs. PsC using our 10-fold ensemble approach (with Shrinkage Discriminant Analysis). The area under the ROC (AUROC) is 0.82.

Supplementary Tables

Supplementary Table 1: Detailed Number of Patients and Markers in each Genetic Cohort

Cohort	Patients						Markers (Genotyped and Well Imputed)						
	PsV	PsA	PsC	USP[a]	Control	Total	Genotyped	1KG Imputed	HRC Imputed	SNP[a]	INDEL[a]	HLA/AA[a]	Total
PsA GWAS	1,430	1,430	NA	NA	1,417	4,277	972,453	13,056,825	16,221,274	17,510,941	1,278,891	1,251	18,791,083
CASP GWAS	1,338	349	639	350	1,370	4,046	438,609	10,586,390	14,764,809	15,759,031	1,063,919	1,247	16,824,197
Kiel GWAS	464	33	269	162	1,135	2,063	504,625	10,504,450	12,318,455	13,315,820	1,077,158	1,236	14,394,214
Genizon GWAS	760	139	399	222	993	2,513	489,501	10,765,816	12,555,720	13,624,904	1,093,913	1,224	14,720,041
Exomechip	3,863	752	1,374	1,737	4,027	11,753	461,092	9,586,593	15,571,069	16,411,455	976,233	1,254	17,388,942
PAGE Immunochip	3,169	971	885	1,313	7,394	13,732	160,228	897,698	1,323,168	1,414,274	84,270	1,245	1,499,789

Cohort	PsV	PsA	PsC	USP[a]	Control	Total
New Total	11,024	3,674	3,566	3,784	16,336	27,360
New GWAS Total	7,855	2,703	2,681	2,471	8,943	16,798
Previous[1] Total	9,293	3,061	3,110	3,122	17,393	26,686
Previous[1] GWAS Total	4,007	1,946	1,363	698	4,934	8,941

	SNP[a]	INDEL[a]	HLA/AA[a]	Total
New Union	23,657,701 (8,730,264[b])	1,403,045 (1,021,305[b])	1,270 (1,217[b])	25,062,016 (9,752,786[b])
New Intersection (All)	1,120,138 (43,356[c])	66,845 (3,301[c])	1,203 (546[c])	1,188,186 (47,203[c])
New Intersection (GWAS)	9,771,987 (247,740[c])	870,338 (27,115[c])	1,205 (546[c])	10,643,530 (275,401[c])
Previous[1,2] Union	8,265,477 (7,091,979[b])	681,304 (627,111[b])	1,342 (1,216[b])	8,948,123 (7,720,306[b])
Previous[1,2] Intersection (All)	40,249 (8,775[c])	3,187 (717[c])	1,141 (309[c])	44,577 (9,801[c])
Previous[1,2] Intersection (GWAS)	6,964,145 (229,722[c])	589,032 (20,195[c])	1,269 (326[c])	7,554,446 (250,243[c])

Abbreviations are as follows: PsV, psoriasis vulgaris; PsA, psoriatic arthritis; PsC, cutaneous-only psoriasis; USP, undefined sub-phenotype; NA, not available. Patients with undefined sub-phenotype (USP) have had a PsV diagnosis within the last 10 years, but have not been diagnosed with PsA (as such, they may have PsA or PsC). [a]Well imputed markers (r^2>=0.7). [b]Union of markers filtered using MAF>=0.01 (these are the markers used in our unconditional meta-analysis). [c]Intersection of markers filtered using MAF>=*0.01* and *p≤0.05* (these are the markers used in our conditional meta-analysis). All the samples are of Caucasian descent, with 49.7% male and 50.3% female psoriasis samples. The mean age at onset for PsV is 28 (SD: 15) and for PsA it is 39 (SD: 13), based on available data at the time of the study.

Supplementary Table 2: Summary of Conditional Meta-Analysis Results

Chr	Including PAGE	Excluding PAGE
1	21	13
2	16	14
3	10	17
4	15	17
5	15	15
6	25	21
7	9	10
8	17	11
9	10	15
10	8	9
11	9	7
12	6	0
13	3	6
14	6	10
15	3	7
16	8	7
17	3	6
18	5	3
19	6	3
20	0	7
21	3	2
22	2	5

Abbreviations are as follows: Chr, chromosome. The counts in the above table represent the number of markers from each chromosome identified through stepwise conditional analysis (out of 200 markers in total), when including or excluding the PAGE Immunochip cohort

Supplementary Table 3: Precision, Recall and Specificity Predicting Different Proportions of Samples as PsA

Percentage of Samples Predicted as PsA	Conditional Analysis (No PAGE)			10-fold Ensemble Approach (No PAGE)		
	Precision (%)	Recall (%)	Specificity (%)	Precision (%)	Recall (%)	Specificity (%)
5%	99.8	16.7	100.0	92.6	15.5	99.5
10%	98.3	33.0	99.8	74.1	24.8	96.3
15%	92.8	46.1	98.5	71.3	35.4	93.9
20%	83.0	55.1	95.1	66.4	44.1	90.4
25%	72.8	60.6	90.3	61.2	50.9	86.1
30%	65.1	65.1	85.0	59.0	59.0	82.4
35%	59.1	69.0	79.5	56.4	65.8	78.1
40%	54.3	72.1	73.9	55.6	73.9	74.7
45%	50.4	75.5	68.1	52.3	78.3	69.3
50%	47.0	78.3	62.2	49.3	82.0	63.7

Supplementary Table 4: Usage and Parameter Settings for Each Machine Learning Algorithm

Algorithm	Usage	Parameter Settings
Random Forest	Highest performing classifier when including PAGE and performing conditional analysis on all data at once	mtry=*sqrt(M)*, nodesize=1, ntree=*500*, replace=TRUE, sampSize=*N*
Conditional Inference Forest	Highest performing classifier when excluding PAGE and performing conditional analysis on all data at once	fraction=*0.632*, minbucket=*7*, minsplit=20, mtry=5, ntree=*500*
Elastic Net	Evaluating an alternative feature selection approach as well as the effect of using more markers	alpha=*0.5*, lambda (estimated automatically[3]), nlambda=*100*
Shrinkage Discriminant Analysis	10-fold ensemble for conditional analysis and model training, to improve robustness on new data	lambda and lambda.var (estimated automatically[4]), diagonal=FALSE

Supplementary Notes

Customized Exomechip Array

The basic Exomechip array contains 246,000 genome-wide markers and 265,000 SNPs/indels from exomes, and 95,000 eQTL, pharmacogenomic and novel loss-of-function variants. This was supplemented by addition of custom markers. For more details, see *Tsoi et al. 2017*[5].

Supplementary References

1 Stuart, P. E. *et al.* Genome-wide association analysis of psoriatic arthritis and cutaneous psoriasis reveals differences in their genetic architecture. *Am. J. Hum. Genet.* **97**, 816-836 (2015).

2 Okada, Y. *et al.* Fine mapping major histocompatibility complex associations in psoriasis and its clinical subtypes. *Am. J. Hum. Genet.* **95**, 162-172 (2014).

3 Friedman, J., Hastie, T. & Tibshirani, R. Regularization paths for generalized linear models via coordinate descent. *J. Stat. Softw.* **33**, doi:10.18637/jss.v033.i01 (2010).

4 Schafer, J. & Strimmer, K. A shrinkage approach to large-scale covariance matrix estimation and implications for functional genomics. *Stat. Appl. Genet. Mol. Biol.* **4**, Article32, doi:10.2202/1544-6115.1175 (2005).

5 Tsoi, L. C. *et al.* Large scale meta-analysis characterizes genetic architecture for common psoriasis-associated variants. *Nat. Commun.*, doi:10.1038/ncomms15382 (2017).
