# Supplementary material for: Genetic signature to provide robust risk assessment of psoriatic arthritis development in psoriasis patients
Source: Nat Commun. 2018 Oct 9;9:4178. doi: 10.1038/s41467-018-06672-6 (PMC6177414; doi:10.1038/s41467-018-06672-6)
Supplement: Supplementary file 2 — Description of Additional Supplementary Files [file 41467_2018_6672_MOESM2_ESM.pdf]

Description of Additional Supplementary Files

File Name: Supplementary Dataset 1
Description: Detailed Conditional Meta-Analysis Results. Abbreviations are as follows: Chr, chromosome. Cond., conditional; Uncond., unconditional. p-values were calculated using the Wald test in PLINK 2.0.

File Name: Supplementary Dataset 2
Description: Detailed 10-fold Conditional Meta-Analysis Results. Abbreviations are as follows: Chr, chromosome. p-values were calculated using the Wald test in PLINK 2.0.
